# Optimal Control and Bifurcation Analysis of HIV Model

**DOI:** 10.1155/2023/4754426

**Published:** 2023-02-06

**Authors:** Kumama Regassa Cheneke

**Affiliations:** ^1^Department of Mathematics, Wollega University, Ethiopia; ^2^Department of Mathematics, Hawassa College of Teacher Education, Ethiopia

## Abstract

In this study, a very crucial stage of HIV extinction and invisibility stages are considered and a modified mathematical model is developed to describe the dynamics of infection. Moreover, the basic reproduction number *R*_0_ is computed using the next-generation matrix method whereas the stability of disease-free equilibrium is investigated using the eigenvalue matrix stability theory. Furthermore, if *R*_0_ ≤ 1, the disease-free equilibrium is stable both locally and globally whereas if *R*_0_ > 1, based on the forward bifurcation behavior, the endemic equilibrium is locally and globally asymptotically stable. Particularly, at the critical point *R*_0_ = 1, the model exhibits forward bifurcation behavior. On the other hand, the optimal control problem is constructed and Pontryagin's maximum principle is applied to form an optimality system. Further, forward fourth-order Runge–Kutta's method is applied to obtain the solution of state variables whereas Runge–Kutta's fourth-order backward sweep method is applied to obtain solution of adjoint variables. Finally, three control strategies are considered and a cost-effective analysis is performed to identify the better strategies for HIV transmission and progression. In advance, prevention control measure is identified to be the better strategy over treatment control if applied earlier and effectively. Additionally, MATLAB simulations were performed to describe the population's dynamic behavior.

## 1. Introduction

Human immunodeficiency virus (HIV) is a virus causing HIV infection and results in economic and life devastating crisis if immediate action is not taken to halt further prevalence of the infection [1–11]. Moreover, this infection has no curing medication, but antiretroviral therapy (ART) or its combination is used for halting further progression of infection by inhibiting the virus in human blood, but if left untreated leads to a sever stage called acquired immunodeficiency syndrome (AIDS) [12–23]. HIV infection progress through stages as follows: (i) primary stage (asymptomatic stage): this stage faces human individual where the virus is in the blood cannot be diagnosed with medical instruments; (ii) asymptomatic stage: this stage is symptomless stage of HIV infection but diagnosable with medical test; (iii) symptomatic stage: in this stage, the symptom of HIV infection like tiredness, loss of weight, and extreme loss of water starts to manifest in the life of HIV infected individuals; and (iv) AIDS stage: this is advanced stage of HIV infection where it is difficult for treatment and leads to death soon if special care is not taken [24–32]. Modes of HIV transmission are through unsafe sexual practices with HIV-infected person, through contacts of normal blood with HIV infected blood, mother to child through breast or birth time, and any contacts of HIV contains fluids of human's with HIV-negative human fluids [33–35]. However, in safe practice, the risk of HIV transmission can be reduced by using principle of abstinence–be faithful–use condom (ABC) [36–38]. Optimal control intervention through public health education, using of condom and treatment benefits a lot for continuing human life in safe [1–3, 7, 11, 15, 19, 25–27, 33, 39–44].

A mathematical model is a crucial scientific representation of both biological and physical problems in the form of mathematical equations [45–52]. Moreover, different mathematical models have been developed to describe the dynamics of HIV with optimal controls. Particularly, a SICA mathematical model of HIV with ART effect on HIV patients is described in [9]. But, a mathematical model of HIV infection transmission with undetectable behavior of individuals has not been studied. Hence, in this study, we are motivated to highlight the significance of adherence of ART with optimal control intervention and cost-effectiveness analysis in a modified HIV model. Further, the effect of early starting of ART that leads to an undetectable level of viral load in the blood of humans is considered and the SWIUA mathematical model of HIV with optimal control is developed. Organization of the paper is follows: In Section 2, we formulate the SWIUA model of HIV that describes the stated assumptions, variables, and parameters and in Section 3, a mathematical analysis of the model without control is carried out. Moreover, in Section 4, optimal control problem is discussed. Finally, in Section 5, numerical simulations, results and discussion, and the conclusion are presented.

## 2. Model Formulation

In this study, a deterministic mathematical model is formulated to analyze the transmission dynamics of the human immunodeficiency virus through applying prevention and treatment control strategies. The current model is a modification of the SIA model of HIV discussed in [13]. A base model has three compartments of human population and did not explicitly show the effectiveness of ART that reduces the viral load in the blood to the undetectable level for ART users. The present work is done to fill the gap observed in the previous study. The compartments of the current model are described as follows:

- Susceptible compartment: this compartment is denoted by *S* and embraces all humans who are free of HIV but have a chance of being infected in the infective environment. All humans in this compartment transfer to the HIV compartment at the transmission rate *β*, provided that effective contact is done with humans in HIV compartment
- HIV untested compartment: this compartment consists of all individuals who are at pre-AIDS stage and not test for HIV. They transmits virus to others at transmission rate *β*_1_
- HIV tested compartment: it is denoted by *I*. This compartment includes all pre-AIDS humans who are infected with HIV and tests positive at healthy center and they transmit virus to susceptible individuals at transmission rate *β*_2_
- Undetectable compartment: it is denoted by *U*. This compartment includes all humans who get infected with human immunodeficiency virus, but with undetectable viral load, as a result of effective usage of antiretroviral treatment
- AIDS compartment: it is denoted by *A*. This compartment includes all humans who are at advanced stage of HIV and face loss of life at disease induced death rate *δ*

In the formulated mathematical model of HIV, the following assumptions are stated:

- The total size of population is assumed to be nonconstant
- It is assumed that HIV untested individuals get tested for HIV infection before they develop AIDS disease
- The total population size at time *t* is denoted by *N*(*t*) is given by

(1) $$\begin{array}{c} N\left(t\right)=S\left(t\right)+W\left(t\right)+I\left(t\right)+U\left(t\right)+A\left(t\right) \end{array}$$

(iv) Susceptible humans are recruited to the compartment *S*(*t*) at some constant rate *τ*
(v) Susceptible humans get HIV and join the HIV compartment at a constant rate *β*
(vi) Individuals transfer from HIV compartment to undetectable compartment at the constant rate *θ* and transfer to AIDS compartment at a constant rate *α*
(vii) Individuals in undetectable compartment transfer to the HIV compartment at a constant rate ∅
(viii) All categories of human's compartments face the same natural mortality with a rate *μ*
(ix) All humans in AIDS compartment suffer disease-induced death at a constant rate *δ*
(x) *u*_1_ is prevention control effort
(xi) *u*_2_ is treatment control effort
(xii) All parameters used in the dynamical system are positive

Moreover, the notations and description of model variables are given in Table 1, whereas model parameters notations and descriptions are given in Table 2.

**Figure 1:**
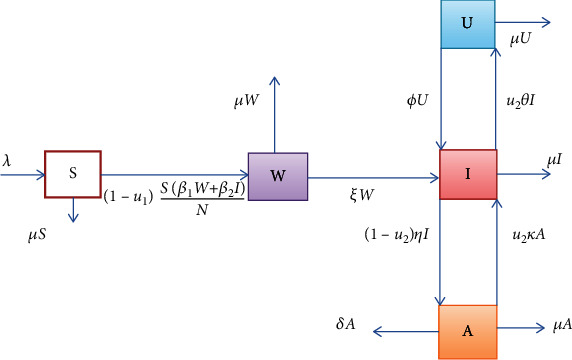
Flow diagram of model in the study.

Based on flow diagram given in Figure 1 and assumptions considered, without control measures, the dynamics of the populations are represented by the subsequent dynamical system:

(2) $$\begin{array}{c} \frac{dS}{dt}=\lambda -\frac{S\left(\beta_{1}W+\beta_{2}I\right)}{N}-\mu S,\\ \frac{dW}{dt}=\frac{S\left(\beta_{1}W+\beta_{2}I\right)}{N}-\left(\xi +\mu \right)W,\\ \frac{dI}{d\text{t}}=\xi W+\phi U-\left(\eta +\mu \right)I,\\ \frac{dU}{dt}=-\left(\emptyset +\mu \right)U,\\ \frac{dA}{dt}=\eta I-\left(\delta +\mu \right)A, \end{array}$$

with nonnegative initial conditions are *S*(0) > 0, *W*(0) ≥ 0, *I*(0) ≥ 0, *U*(0) ≥ 0, *A*(0) ≥ 0.

## 3. Mathematical Analysis of the Model

### 3.1. Invariant Region

**Theorem 1 .**

*Ω* ⊂ *ℛ*_+_^5^ is a region in which all solutions of the model (2) are bounded provided that initial conditions are bounded. That is,

(3) $$\begin{array}{c} \Omega =\left\{\left(S\left(t\right),\;W\left(t\right),\;I\left(t\right),\;U\left(t\right),\;A\left(t\right)\right)\in R_{+}^{5}:N\left(t\right)\leq \frac{\lambda }{\mu }\;\right\}. \end{array}$$

**Proof**

Consider a total population size, *N*(*t*), at time *t* given by

(4) $$\begin{array}{c} N\left(t\right)=S\left(t\right)+W\left(t\right)+I\left(t\right)+U\left(t\right)+A\left(t\right). \end{array}$$

Now, differentiating both sides of equation (4) with respect to time *t*, we have

(5) $$\begin{array}{c} \frac{dN}{dt}=\frac{dS}{dt}+\frac{dW}{dt}+\frac{dI}{dt}+\frac{dU}{dt}+\frac{dA}{dt} \end{array}$$

Reduced to,

(6) $$\begin{array}{c} \frac{dN}{dt}=\lambda -\mu N-\delta A\leq \lambda -\mu N\Rightarrow \frac{dN}{dt}\leq \lambda -\mu \Rightarrow \frac{dN}{\tau -\mu N}\leq dt. \end{array}$$

Integrating both sides of (6) and using comparison theorem [53], we have,

(7) $$\begin{array}{c} N\left(t\right)\leq \frac{\lambda }{\mu }-\left(\frac{\lambda }{\mu }-N\left(0\right)\right)e^{-\mu t}. \end{array}$$

Here, from (7) it follows that as *t*⟶∞, *N*(*t*)⟶*λ*/*μ*. That is, for all possibility, the expressions on the right hand side of the inequality (7) either increase to least upper bound or decrease to greatest lower bound as time increase. Hence, *N*(*t*) ≤ *λ*/*μ*.

Therefore, the feasible solution set of model (2) is the invariant region *Ω*, defined as

(8) $$\begin{array}{c} \Omega =\left\{\left(\begin{array}{c} S\left(t\right),\;W\left(t\right),\;I\left(t\right),\;U\left(t\right),\;A\left(t\right) \end{array}\right)\in R_{+}^{5}:N\left(t\right)\leq \frac{\lambda }{\mu }\;\right\}. \end{array}$$

### 3.2. Positivity of Model Solutions

**Theorem 2 .**

Solutions of the model (2) are always nonnegative for all *t* and will remain in ℝ_+_^5^.

**Proof**

The proof follows by showing that each solution variable is nonnegative. Considering the first equation of model (2) we have

(9) $$\begin{array}{c} \frac{dS}{dt}=\lambda -\frac{S\left(\beta_{1}W+\beta_{2}I\right)}{N}-\mu S. \end{array}$$

Ignoring the positive term *λ* from the preceding equation we get the following inequality.

(10) $$\begin{array}{c} \frac{dS}{S}\geq -\left(\frac{\beta_{1}W+\beta_{2}I}{N}\right)dt. \end{array}$$

Solving the forgoing inequality over time interval [0, *t*], we get the following inequality.

(11) $$\begin{array}{c} S\left(t\right)\geq S\left(0\right)e^{-\mu t-\int_{0}^{t\beta_{1}W\left(\xi \right)+\beta_{2}I\left(\xi \right)/N\left(\xi \right)}d\xi }. \end{array}$$

In the preceding inequality, we know that *e*^−*μt*−∫_0_^*t*^(*β*_1_*W*(*ξ*) + *β*_2_*I*(*ξ*)/*N*(*ξ*))*dξ*^ is nonnegative exponential expression, and *S*(0) is positive from initial condition. Therefore, *S*(*t*) is nonnegative for all time *t*. Similarly, the remaining solution variables *W*(*t*), *I*(*t*), *U*(*t*) and *A*(*t*) are nonnegative. Hence, the solutions of model (2) are nonnegative.

Therefore, from the discussions in subsection it can be concluded that the formulated model is mathematically well-posed and solutions of the model are biologically meaningful.

### 3.3. Existence and Uniqueness of Solutions

**Theorem 3 .**

The solutions of model (2) exists and unique.

**Proof**

Applying Theorem 1, all expressions on the right hand of equality of model (2) are continuously differentiable and bounded. Therefore, by Cauchy-Lipchitz theorem we can conclude that the formulated model has unique solution for all positive time *t*.

### 3.4. Equilibria of Model

#### 3.4.1. Disease-Free Equilibrium (DFE)

The disease-free equilibrium point of model (2) is a steady state point where there is no HIV infection in the population. Hence, to obtain DFE the state variables *I*, *A*, and *U* are set to zero (*W* = *I* = *A* = *U* = 0). The computed disease–free equilibrium point *E*_0_ of the model (2) is given by

(12) $$\begin{array}{c} E_{0}=\left(\begin{array}{c} \frac{\lambda }{\mu },\;0,\;0,\;0,\;0 \end{array}\right). \end{array}$$

#### 3.4.2. Endemic Equilibrium (EE)

An endemic equilibrium point is steady state point where disease persists in the population. The endemic equilibrium is obtained by setting the right-hand of model equation to zero and evaluating for *S*, *W*, *I*, *U*, and *A* in terms of model parameters. Moreover, adding equations (2) and (4), we obtain *I* in terms of *S*. Finally, using equation (4), setting *N* = *S* + *W* + *I* + *U* + *A*, and expressing all variables in terms of *S*, we obtain the desired solution. Thus, the computed endemic equilibrium *E*^1^ = (*S*^∗^, *W*^∗^, *I*^∗^, *U*^∗^, *A*^∗^) of model (2) is given by

(13) $$\begin{array}{c} E^{1}=\left(\frac{\lambda \left(1+d+bd\right)}{\beta_{1}+\beta_{2}d+\mu d+b\mu d-\xi },\;W^{*},\;dW^{*},\;\;0,\;\;bI^{*}\right), \end{array}$$

where

(14) $$\begin{array}{c} W^{*}=\frac{\lambda \left(R_{0}-1\right)}{\beta_{1}+\beta_{2}d+\mu d+b\mu d-\xi },\;b=\frac{\eta }{\delta +\mu },\;d=\frac{\xi }{\eta +\mu }. \end{array}$$

### 3.5. Basic Reproduction Number

The basic reproduction number is the average number of cases generated by one infected person in wholly susceptible population [45–50]. The basic reproductive number *R*_0_ can be determined using the next-generation matrix. In this method, *R*_0_ is defined as the largest eigenvalue of the next-generation matrix [51]. The formulation of this matrix involves classification of all compartments of the model in to two classes: infected and noninfected. Let *f* be a matrix of newly infected cases and *v* be a matrix of transition cases in model (2). Consider model (2).

Now *f* and *v* are given, respectively, as,

(15) $$\begin{array}{c} f=\begin{array}{cc} \;\left[\begin{array}{c} \frac{S\left(\beta_{1}W+\beta_{2}I\right)}{N}\\ 0\\ 0\\ 0 \end{array}\right], & \;v=\left[\begin{array}{c} \left(\xi +\mu \right)W\\ -\xi W-\phi U+\left(\eta +\mu \right)I\\ \left(\emptyset +\mu \right)U\\ -\eta I+\left(\delta +\mu \right)A \end{array}\right] \end{array}, \end{array}$$

The Jacobian of *f* and *v* evaluated at disease-free equilibrium point *E*_0_ is given by *F* and *V*, respectively, as follows,

(16) $$\begin{array}{c} F=\begin{array}{cc} \left[\begin{array}{cccc} \beta_{1} & \beta_{2} & 0 & 0\\ 0 & 0 & 0 & 0\\ 0 & 0 & 0 & 0\\ 0 & 0 & 0 & 0 \end{array}\right], & V=\left[\begin{array}{cccc} \xi +\mu & 0 & 0 & 0\\ -\xi & \eta +\mu & -\phi & 0\\ 0 & 0 & \emptyset +\mu & 0\\ 0 & -\eta & 0 & \delta +\mu \end{array}\right] \end{array}. \end{array}$$

The next-generation matrix, *FV*^−1^, is computed as

(17) $$\begin{array}{c} FV^{-1}=\left[\begin{array}{cccc} \beta_{1} & \beta_{2} & 0 & 0\\ 0 & 0 & 0 & 0\\ 0 & 0 & 0 & 0\\ 0 & 0 & 0 & 0 \end{array}\right]{\left[\begin{array}{cccc} \xi +\mu & 0 & 0 & 0\\ -\xi & \left(\eta +\mu \right) & -\phi & 0\\ 0 & 0 & \emptyset +\mu & 0\\ 0 & -\eta & 0 & \delta +\mu \end{array}\right]}^{-1}=\left[\begin{array}{cccc} \frac{\beta_{1}}{\xi +\mu }+\frac{\beta_{2}\xi }{\left(\xi +\mu \right)\left(\eta +\mu \right)}\; & \frac{\beta_{2}}{\eta +\mu } & \frac{\beta_{2}\phi }{\left(\phi +\mu \right)\left(\eta +\mu \right)} & 0\\ 0 & 0 & 0 & 0\\ 0 & 0 & 0 & 0\\ 0 & 0 & 0 & 0 \end{array}\right]. \end{array}$$

The eigenvalues of next-generation matrix are computed and given by

(18) $$\begin{array}{c} \lambda_{1}=\frac{\beta_{1}}{\xi +\mu }+\frac{\beta_{2}\xi }{\left(\xi +\mu \right)\left(\eta +\mu \right)},\;\lambda_{2}=0,\;\lambda_{3}=0,\;\lambda_{4}=0. \end{array}$$

Here, *λ*_1_ is the largest eigenvalue of next-generation matrix. Therefore, the reproduction number, *R*_0_, is given by

(19) $$\begin{array}{c} R_{0}=\frac{\beta_{1}}{\xi +\mu }+\frac{\beta_{2}\xi }{\left(\xi +\mu \right)\left(\eta +\mu \right)}. \end{array}$$

### 3.6. Bifurcation Analysis

The Hopf bifurcation is studied in [5], and it describes the behavior of the system as system changes its steady state. In this subsection, we verify the possibility of backward and forward bifurcation using the center manifold theory stated in reference [28]. Now, model (2) can be written in the vector form, by renaming the variables as *S* = *x*_1_, *W* = *x*_2_, *I* = *x*_3_, *U* = *x*_4_, *A* = *x*_5_. That is,

(20) $$\begin{array}{c} \frac{dX}{dt}=F\left(X\right), \end{array}$$

where, $X={\left(\begin{array}{c} x_{1},x_{2}x_{3},x_{4}x_{5}, \end{array}\right)}^{T},F\left(X\right)={\left(\begin{array}{c} f_{1}f_{2},f_{3},f_{4},f_{5} \end{array}\right)}^{T}$. Then, model (2) becomes

(21) $$\begin{array}{c} \frac{dx_{1}}{dt}=\lambda -\frac{\left(\beta_{1}x_{1}x_{2}+\beta_{2}x_{1}x_{3}\right)}{N}-\mu x_{1}=f_{1},\\ \frac{dx_{2}}{dt}=\frac{\left(\beta_{1}x_{1}x_{2}+\beta_{2}x_{1}x_{3}\right)}{N}-\left(\xi +\mu \right)x_{2}=f_{2},\\ \frac{dx_{3}}{dt}=\xi x_{2}+\phi x_{4}-\left(\eta +\mu \right)x_{3}=f_{3},\\ \frac{dx_{4}}{dt}=-\left(\emptyset +\mu \right)x_{4}=f_{4},\\ \frac{dx_{5}}{dt}=\eta x_{3}-\left(\delta +\mu \right)x_{5}=f_{5}. \end{array}$$

Here, from preceding system of nonlinear equation, choosing *β*_1_ as a bifurcation parameter and setting *R*_0_ = 1, we have

(22) $$\begin{array}{c} \beta_{1}^{*}\equiv \beta_{1}=\xi +\mu -\frac{\beta_{2}\xi }{\eta +\mu }. \end{array}$$

So that the disease-free equilibrium, *E*_0_, is locally stable when *β*_1_ < *β*_1_^∗^ and is unstable when *β*_1_ > *β*_1_^∗^. The linearized matrix of the system around the disease-free equilibrium *E*_0_ and evaluated at *β*_1_^∗^ is given by

(23) $$\begin{array}{c} J\left(\begin{array}{cc} E_{0}, & \beta_{1}^{*} \end{array}\right)=\left[\begin{array}{ccccc} -\mu & -\left(\xi +\mu \right)+\frac{\beta_{2}\xi }{\eta +\mu } & -\beta_{2} & 0 & 0\\ 0 & -\frac{\beta_{2}\xi }{\eta +\mu } & \beta_{2} & 0 & 0\\ 0 & \xi & -\left(\eta +\mu \right) & \phi & 0\\ 0 & 0 & 0 & -\left(\emptyset +\mu \right) & 0\\ 0 & 0 & \eta & 0 & -\left(\delta +\mu \right) \end{array}\right]. \end{array}$$

The eigenvalues of matrix $J\left(\begin{array}{c} E_{0},\beta_{1}^{*} \end{array}\right)$ are *λ*_1_ = 0, *λ*_2_ = −*μ*, *λ*_3_ = −*δ* − *μ*. The sign of remaining eigenvalues are determined from characteristic polynomial:

(24) $$\begin{array}{c} f\left(\lambda \right)=a_{0}\lambda^{2}+a_{1}\lambda +a_{2}, \end{array}$$

where

(25) $$\begin{array}{c} a_{0}=1,\\ a_{1}=\frac{\phi^{2}\eta +\phi^{2}\mu +\phi \eta^{2}+4\phi \eta \mu +3\phi \mu^{2}+\beta_{2}\xi \phi +\eta^{2}\mu +3\eta \mu^{2}+2\mu^{3}+\beta_{2}\xi \mu }{\phi \eta +\phi \mu +\eta \mu +\mu^{2}},\\ a_{2}=\frac{\phi^{2}\eta^{2}+\beta_{2}\xi \phi^{2}+2\phi \mu \left(\phi \eta +\eta^{2}+2\eta \mu +\mu^{2}+\beta_{2}\xi \right)+\left(\phi^{2}+\eta^{2}+2\eta \mu +\mu^{2}+\beta_{2}\xi \right)\mu^{2}\;}{\phi \eta +\phi \mu +\eta \mu +\mu^{2}}. \end{array}$$

We observe that coefficients, *a*_0_, *a*_1_, and *a*_2_, are positive, and all eigenvalues of Jacobian matrix are negative while one eigenvalue is zero. Moreover, let $w={\left(\begin{array}{c} w_{1},w_{2},w_{3},w_{4},w_{5} \end{array}\right)}^{T}$ be the right eigenvector, associated with simple zero eigenvalue and can be obtained by solving equation $J\left(\begin{array}{c} E_{0},\beta^{*} \end{array}\right)w=0$. Thus, we have

(26) $$\begin{array}{c} w_{1}=-\left(\xi +\mu \right)\frac{\left(\eta +\mu \right)\left(\phi +\mu \right)+\theta \mu }{\xi \mu \left(\phi +\mu \right)}w_{3},w_{2}=\frac{\eta +\mu }{\xi }w_{3},w_{3}=w_{3},w_{4}=\frac{\theta w_{3}}{\phi +\mu },w_{5}=\frac{\eta w_{3}}{\delta +\mu }. \end{array}$$

Also, let $v=\left(\begin{array}{c} v_{1},v_{2},v_{3},v_{4},v_{5} \end{array}\right)$ be the left eigenvector corresponding to simple zero eigenvalue, obtained by setting and solving $vJ\left(\begin{array}{c} E_{0},\beta^{*} \end{array}\right)=0$. This gives, *v*_1_ = 0, *v*_2_ = (*η* + *μ*)/*β*_2_*v*_3_, *v*_3_ = *v*_3_, *v*_4_ = *ϕv*_3_/(*ϕ* + *μ*), *v*_5_ = 0. In order that the required conditions *v*.*w* = 1, the product gives,

(27) $$\begin{array}{c} v_{1}w_{1}+v_{2}w_{2}+v_{3}w_{3}+v_{4}w_{4}+v_{5}w_{5}=1. \end{array}$$

Again substituting the corresponding components in the preceding equation, we have

(28) $$\begin{array}{c} v_{3}w_{3}\frac{{\left(\eta +\mu \right)}^{2}+\beta_{2}\xi }{\beta_{2}\xi }=1. \end{array}$$

Thus, the preceding equality is satisfied if we choose

(29) $$\begin{array}{c} v_{3}=\beta_{2}\xi ,\;w_{3}=\frac{1}{{\left(\eta +\mu \right)}^{2}+\beta_{2}\xi }. \end{array}$$

Next, we compute all second-order partial derivatives of functions on the right hand of (∗) as given in the appendix.

Next, we compute bifurcation coefficients *a* and *b*:

(30) $$\begin{array}{c} a=\overset{4}{\underset{k,i,j=1}{\sum }}v_{k}w_{i}w_{j}\frac{\partial^{2}f_{k}}{\partial x_{i}\partial x_{j}}\left(E_{0},\beta^{*}\right)=\overset{4}{\underset{i,j=1}{\sum }}v_{2}w_{i}w_{j}\frac{\partial^{2}f_{2}}{\partial x_{i}\partial x_{j}}\left(E_{0},\beta^{*}\right)=-\frac{2\beta_{1}}{x_{1}}-\frac{\beta_{1}}{x_{1}}-\frac{\beta_{2}}{x_{1}}-\frac{\beta_{1}}{x_{1}}-\frac{2\beta_{2}}{x_{1}}-\frac{\beta_{2}}{x_{1}}<0,\\ b=\overset{4}{\underset{k,i=1}{\sum }}v_{k}w_{i}\frac{\partial^{2}f_{k}}{\partial x_{i}\partial \beta^{*}}\left(E_{0},\beta^{*}\right)=v_{2}w_{2}=\frac{{\left(\eta +\mu \right)}^{2}}{{\left(\eta +\mu \right)}^{2}+\beta_{2}\rho }>0. \end{array}$$

Since *a* < 0 and *b* > 0, the model exhibits forward bifurcation at *R*_0_ = 1.

Next, the following procedures given in (Biswas et al., 2020), we compute the bifurcation coefficients *a* and *b*, to identify the direction of bifurcation at *R*_0_ = 1. Thus, we have

(31) $$\begin{array}{c} a=\overset{4}{\underset{k,i,j=1}{\sum }}v_{k}w_{i}w_{j}\frac{\partial^{2}f_{k}}{\partial x_{i}\partial x_{j}}\left(E_{0},\beta^{*}\right)=-\left(\frac{2v_{2}\mu^{3}w_{2}^{2}\beta^{*}}{\tau^{3}}+\frac{2v_{2}w_{2}\mu \beta^{*}}{\tau \left({\left(\phi +\mu \right)}^{2}\right)}\right),\\ b=\overset{4}{\underset{k,i=1}{\sum }}v_{k}w_{i}\frac{\partial^{2}f_{k}}{\partial x_{i}\partial \beta^{*}}\left(E_{0},\beta^{*}\right)=v_{2}w_{2}. \end{array}$$

Since all parameters in model (2) are nonnegative and additionally *v*_2_ and *w*_2_ are positive, we conclude *a* < 0 and *b* > 0. Thus, according to [5], model (2) exhibits a supercritical (forward) bifurcation, when *R*_0_ crosses the threshold *R*_0_ = 1. That is, there exist locally asymptotically stable endemic equilibrium point $E_{1}=\left(\begin{array}{c} S^{*},W^{*},I^{*},U^{*},A^{*} \end{array}\right)$ for *R*_0_ > 1. Based on the results of the above discussion and [7], the following theorem is stated.

**Theorem 4 .**

The *trans*-critical bifurcation of model (2) that occurs at *R*_0_ = 1 is a supercritical (forward) bifurcation. That is, there exists locally asymptotically stable endemic equilibrium point $E_{1}=\left(\begin{array}{c} S^{*},W^{*},I^{*},U^{*},A^{*} \end{array}\right)$ for *R*_0_ > 1.

**Remark 1 .**

Theorem 4 shows that if *R*_0_ > 1, then a few inflow of infectious individuals in fully susceptible population can result in persistence of the HIV in the population.

### 3.7. Stability Analysis of Equilibrium Points

In absence of the infectious disease, model (2) has a unique disease-free steady-state *E*_0_, and in the presence of disease, model (2) has unique endemic equilibrium *E*_1_.

#### 3.7.1. Local Stability of Disease-Free Equilibrium

**Theorem 5 .**

The DFE *E*_0_ of the model (2) is locally asymptotically stable if *R*_0_ < 1 and unstable if *R*_0_ > 1.

**Proof**

Consider model (2), so that the Jacobian matrix of the system at DFE is given by

(32) $$\begin{array}{c} J=\left[\begin{array}{ccccc} -\mu & -\beta_{1} & -\beta_{2} & 0 & 0\\ 0 & \beta_{1}-\left(\xi +\mu \right) & \beta_{2} & 0 & 0\\ 0 & \xi & -\left(\eta +\mu \right) & \phi & 0\\ 0 & 0 & 0 & -\left(\emptyset +\mu \right) & 0\\ 0 & 0 & \eta & 0 & -\left(\delta +\mu \right) \end{array}\right]. \end{array}$$

The eigenvalues of a preceding Jacobian matrix *J* is computed and given by

(33) $$\begin{array}{c} \lambda_{1}=-\mu ,\lambda_{2}=-\left(\delta +\mu \right),\lambda_{3}=-\left(\emptyset +\mu \right),\lambda_{4}=\frac{\beta_{1}-\xi -\eta -2\mu -\sqrt{{\left(\beta_{1}-\xi \right)}^{2}+2\eta \beta_{1}-2\xi \eta +4\beta_{2}\xi +\eta^{2}}}{2},\lambda_{5}=\frac{\beta_{1}-\xi -\eta -2\mu +\sqrt{{\left(\beta_{1}-\xi \right)}^{2}+2\eta \beta_{1}-2\xi \eta +4\beta_{2}\xi +\eta^{2}}}{2}. \end{array}$$

Clearly, the first three eigenvalues are negative whereas the fourth and fifth eigenvalues are negative if *R*_0_ < 1.

Further, by a stability analysis of a point using Jacobian matrix, we conclude that the disease-free equilibrium point is locally asymptotically stable if *R*_0_ < 1 and unstable otherwise.

#### 3.7.2. Local Stability of Endemic Equilibrium Point

**Theorem 6 .**

The EE *E*_1_ of the model (2) is locally asymptotically stable if *R*_0_ > 1 and unstable if *R*_0_ < 1. (Proof. Behavior of forward bifurcation.)

#### 3.7.3. Global Stability of Disease–Free Equilibrium Point

To show global stability of disease-free equilibrium *E*_0_, we use the technique employed in [4]. Accordingly, let *X* ∈ ℝ^1^ denote individuals in uninfected compartment (*S*) and *Y* ∈ ℝ^4^ denotes individuals in infected compartments $\left(\begin{array}{c} W,I,U,A \end{array}\right)\;$. Hence, we write model (2) in the form:

(34) $$\begin{array}{c} \frac{dX}{dt}=H\left(X,Y\right),\\ \frac{dY}{dt}=G\left(X,Y\right),G\left(X,0\right)=0. \end{array}$$

Also, the disease-free equilibrium is given by

(35) $$\begin{array}{c} E_{0}^{1}=\left(X^{0},0\right). \end{array}$$

Here, *X*^0^ is the disease-free equilibrium of the foregoing system.

To guarantee global asymptotic stability of the disease-free equilibrium point, the technique we employed must met the following two conditions H1 and H2.

H1: for *dX*/*dt* = *H*(*X*, 0), *X*^0^ is globally asymptotically stable

H2: $\text{G}\left(X,Y\right)=PY-\hat{G}\left(X,Y\right),\hat{G}\left(X,Y\right)\geq 0$ for (*X*, *Y*) ∈ *Ω*

Here, *P* = *D*_*Y*_*G*(*X*, 0) is a Metzler matrix and *Ω* is a region where solutions of the model are biologically feasible.

**Theorem 7 .**

The disease-free equilibrium point *E*_0_ of model (2) is globally asymptotically stable in a region *Ω* if *R*_0_ < 1 as the fourth and fifth eigenvalues are negative if *R*_0_ < 1 and unstable whenever *R*_0_ > 1 provided that the above two conditions H1 and H2 are satisfied, where *Ω* is a feasible solution region of model (2) in ℝ_+_^5^.

**Proof**

From model (2), we have

(36) $$\begin{array}{c} H\left(X,0\right)=\lambda -\mu S=H\left(S,0\right). \end{array}$$

Putting *H*(*X*, 0) = 0 and solving, we obtain *S* = *λ*/*μ*. Hence, *X*^0^ = (*λ*/*μ*, 0). Clearly, *X*^0^ is globally asymptotically stable equilibrium point of equation:

(37) $$\begin{array}{c} \frac{dX}{dt}=H\left(X,0\right). \end{array}$$

From infected compartments of model (2), we have

(38) $$\begin{array}{c} G\left(X,Y\right)=\left[\begin{array}{cccc} \frac{\beta_{1}S}{N}-\left(\xi +\mu \right) & \frac{\beta_{2}S}{N} & 0 & 0\\ \xi & -\left(\eta +\mu \right) & \phi & 0\\ 0 & 0 & -\left(\emptyset +\mu \right) & 0\\ 0 & \eta & 0 & -\left(\delta +\mu \right) \end{array}\right]\left[\begin{array}{c} W\\ I\\ U\\ A \end{array}\right]=\left[\begin{array}{cccc} \frac{\beta_{1}S}{N} & \frac{\beta_{2}S}{N} & 0 & 0\\ \xi & -\left(\eta +\mu \right) & \phi & 0\\ 0 & 0 & -\left(\emptyset +\mu \right) & 0\\ 0 & \eta & 0 & -\left(\delta +\mu \right) \end{array}\right]\left[\begin{array}{c} W\\ I\\ U\\ A \end{array}\right]-\left[\begin{array}{cccc} \left(\xi +\mu \right) & 0 & 0 & 0\\ 0 & 0 & 0 & 0\\ 0 & 0 & 0 & 0\\ 0 & 0 & 0 & 0 \end{array}\right]\left[\begin{array}{c} W\\ I\\ U\\ A \end{array}\right]. \end{array}$$

In the preceding computation, let

(39) $$\begin{array}{c} P=\left[\begin{array}{cccc} \frac{\beta_{1}S}{N} & \frac{\beta_{2}S}{N} & 0 & 0\\ \xi & -\left(\eta +\mu \right) & \phi & 0\\ 0 & 0 & -\left(\emptyset +\mu \right) & 0\\ 0 & \eta & 0 & -\left(\delta +\mu \right) \end{array}\right], \end{array}$$

so that at disease-free equilibrium it reduces to

(40) $$\begin{array}{c} P=\left[\begin{array}{cccc} \beta_{1}-\left(\xi +\mu \right) & \beta_{2} & 0 & 0\\ \xi & -\left(\eta +\mu \right) & \phi & 0\\ 0 & 0 & -\left(\emptyset +\mu \right) & 0\\ 0 & \eta & 0 & -\left(\delta +\mu \right) \end{array}\right]. \end{array}$$

Again, *PY* gives

(41) $$\begin{array}{c} PY=\left[\begin{array}{cccc} \beta_{1}-\left(\xi +\mu \right) & \beta_{2} & 0 & 0\\ \xi & -\left(\eta +\mu \right) & \phi & 0\\ 0 & 0 & -\left(\emptyset +\mu \right) & 0\\ 0 & \eta & 0 & -\left(\delta +\mu \right) \end{array}\right]\left[\begin{array}{c} W\\ I\\ U\\ A \end{array}\right]. \end{array}$$

Let

(42) $$\begin{array}{c} \hat{G}\left(X,Y\right)=PY-G=\left[\begin{array}{cccc} \beta_{1}-\left(\xi +\mu \right) & \beta_{2} & 0 & 0\\ \xi & -\left(\eta +\mu \right) & \phi & 0\\ 0 & 0 & -\left(\emptyset +\mu \right) & 0\\ 0 & \eta & 0 & -\left(\delta +\mu \right) \end{array}\right]\left[\begin{array}{c} W\\ I\\ U\\ A \end{array}\right]-\left[\begin{array}{cccc} \frac{\beta_{1}S}{N}-\left(\xi +\mu \right) & \frac{\beta_{2}S}{N} & 0 & 0\\ \xi & -\left(\eta +\mu \right) & \phi & 0\\ 0 & 0 & -\left(\emptyset +\mu \right) & 0\\ 0 & \eta & 0 & -\left(\delta +\mu \right) \end{array}\right]\left[\begin{array}{c} W\\ I\\ U\\ A \end{array}\right]. \end{array}$$

Therefore,

(43) $$\begin{array}{c} \hat{G}\left(X,Y\right)=PY-G=\left[\begin{array}{c} \beta_{1}W+\beta_{2}I-\frac{S\left(\beta_{1}W+\beta_{2}I\right)}{N}\\ 0\\ 0\\ 0 \end{array}\right]. \end{array}$$

This implies that

(44) $$\begin{array}{c} \hat{G}\left(X,Y\right)=\left[\begin{array}{c} \left(\beta_{1}W+\beta_{2}I\right)\left(1-\frac{S}{S+W+I+U+A}\right)\\ 0\\ 0\\ 0 \end{array}\right]. \end{array}$$

Since *S* ≤ *S* + *W* + *I* + *U* + *A*, we have (*β*_1_*W* + *β*_2_*I*)(1 − *S*/(*S* + *W* + *I* + *U* + *A*)) ≥ 0; the preceding matrix is a nonnegative matrix. Therefore, it can be written as

(45) $$\begin{array}{c} \hat{G}\left(X,Y\right)=\left[\begin{array}{c} \left(\beta_{1}W+\beta_{2}I\right)\left(1-\frac{S}{S+W+I+U+A}\right)\\ 0\\ 0\\ 0 \end{array}\right]\geq 0. \end{array}$$

Clearly, the second condition is satisfied as $G\left(X,Y\right)=PY-\hat{G}\left(X,Y\right)$, where, $\hat{G}\left(X,Y\right)\geq 0,\forall X,Y$ in the invariant region. Comparing the computations, we have

(46) $$\begin{array}{c} P=\left[\begin{array}{cccc} \beta_{1}-\left(\xi +\mu \right) & \beta_{2} & 0 & 0\\ \xi & -\left(\eta +\mu \right) & \phi & 0\\ 0 & 0 & -\left(\emptyset +\mu \right) & 0\\ 0 & \eta & 0 & -\left(\delta +\mu \right) \end{array}\right]=D_{Y}G\left(X,0\right), \end{array}$$

which is a Metzler matrix as all off diagonal elements of a matrix are nonnegative.

Therefore, the disease-free equilibrium *E*_0_ is globally asymptotically stable if *R*_0_ < 1 as the fourth and fifth eigenvalues are negative if *R*_0_ < 1.

### 3.8. Sensitivity Analysis

Sensitivity analysis is used to determine the sensitivity of the variable with respect to the parameters involved in it [8, 13, 28]. The normalized forward sensitivity index of a particular variable, *R*, with respect to a parameter, *p*, is defined as

(47) $$\begin{array}{c} Y_{p}^{R}=\frac{\partial R}{\partial p}\times \frac{p}{R}. \end{array}$$

It is already shown that the explicit expression of *R*_0_ is given by

(48) $$\begin{array}{c} R_{0}=\frac{\beta_{1}}{\xi +\mu }+\frac{\beta_{2}\xi }{\left(\xi +\mu \right)\left(\eta +\mu \right)}. \end{array}$$

The normalized forward sensitivity indices of *R*_0_ with respect to parameters in it are given by

(49) $$\begin{array}{c} Y_{\beta_{1}}^{R_{0}}=\frac{\partial R_{0}}{\partial \beta_{1}}\frac{\beta_{1}}{R_{0}}=\frac{1}{\xi +\mu }\frac{\beta_{1}}{R_{0}},\\ Y_{\beta_{2}}^{R_{0}}=\frac{\partial R_{0}}{\partial \beta_{2}}\frac{\beta_{2}}{R_{0}}=\frac{\xi }{\left(\xi +\mu \right)\left(\eta +\mu \right)}\frac{\beta_{2}}{R_{0}},\\ Y_{\xi }^{R_{0}}=\frac{\partial R_{0}}{\partial \xi }\times \frac{\xi }{R_{0}}=\left(\frac{-\beta_{1}}{{\left(\xi +\mu \right)}^{2}}+\frac{\beta_{2}\mu }{{\left(\xi +\mu \right)}^{2}\left(\eta +\mu \right)}\right)\frac{\xi }{R_{0}},\\ Y_{\mu }^{R_{0}}=\frac{\partial R_{0}}{\partial \mu }\times \frac{\mu }{R_{0}}=\left(\frac{-\beta_{1}}{{\left(\xi +\mu \right)}^{2}}+\frac{-\beta_{2}\xi \left(\eta +\xi +2\mu \right)}{{\left(\xi +\mu \right)}^{2}{\left(\eta +\mu \right)}^{2}}\right)\frac{\mu }{R_{0}},\\ Y_{\eta }^{R_{0}}=\frac{\partial R_{0}}{\partial \eta }\times \frac{\eta }{R_{0}}=\frac{-\beta_{2}\xi }{{\left(\xi +\mu \right)\left(\eta +\mu \right)}^{2}}\frac{\eta }{R_{0}}, \end{array}$$

where, based on parametric values given in Table 3, we obtain

(50) $$\begin{array}{c} R_{0}=8.23. \end{array}$$

From Table 3, it can be observed that parameters *β*_1_ and *β*_2_ have positive sensitivity indices and the values of the remaining two parameters *ξ*, *η*, and *μ* get negative sensitivity indices. Further, these parameters affect the value of a reproduction number that helps in the analysis of virus extinction or persistence of the disease in the population. On the other hand, an increase of positive parameter value will increase the value of *R*_0_; this implies that disease transmission increase with significant amount. Also, an increase in the magnitude of negative parameter value will cause a value of reproduction number to decrease in some amount, which means the disease transmission significantly decreases in some amount.

## 4. Optimal Control Problem

In this section, we extend SWIUA model by considering control strategies. The optimal control analysis assists to identify the best control strategies to eradicate or control the disease in the community at a specified period of time [3, 15, 19]. In the optimal control problem, the following three control measures are used.

- Preventive control measure *u*_1_ that protects susceptible population from getting the disease
- Treatment control measure *u*_2_ that is used by patients to reduce viral load in the body and slow/stop the progression of the virus

Now, including these control measures *u*_1_ and *u*_2_ in model (2), we get the following optimal control model:

(51) $$\begin{array}{c} \frac{dS}{dt}=\lambda -\left(1-u_{1}\right)\frac{S\left(\beta_{1}W+\beta_{2}I\right)}{N}-\mu S,\\ \frac{dW}{dt}=\left(1-u_{1}\right)\frac{S\left(\beta_{1}W+\beta_{2}I\right)}{N}-\left(\xi +\mu \right)W,\\ \frac{dI}{dt}=\xi W+u_{2}\kappa A+\phi U-\left(\left(1-u_{2}\right)\eta +u_{2}\theta +\mu \right)I,\\ \frac{dU}{dt}=u_{2}\theta I-\left(\emptyset +\mu \right)U,\\ \frac{dA}{dt}=\left(1-u_{2}\right)\eta I-\left(u_{2}kA+\delta +\mu \right)A, \end{array}$$

with nonnegative initial conditions

To study optimal levels of the controls, we define the Lebesgue measurable control set *U* as follows:

(52) $$\begin{array}{c} U=\left\{\left(u_{1}\left(t\right),u_{2}\left(t\right)\right)\text{: }0\leq u_{1}\leq 1,0\leq u_{2}\leq 1,0\leq t\leq t_{f}\right\}. \end{array}$$

Our goal is to find the optimal controls *u*_1_^∗^, and *u*_2_^∗^ optimal solutions *S*^∗^, *W*^∗^, *I*^∗^, *U*^∗^, and *A*^∗^ by fixing the terminal time *t*_*f*_ that minimize the objective functional *J* given by

(53) $$\begin{array}{c} J\left(u_{1},u_{2},u_{3}\right)=\underset{\left(u_{1},u_{2}\right)}{\min}\int_{0}^{t_{f}}c_{1}W+c_{2}I+c_{3}A+\frac{1}{2}\left(w_{1}u_{1}^{2}+w_{2}u_{2}^{2}\right)dt, \end{array}$$

where *c*_1_, *c*_2_, *w*_1_, and *w*_2_ are constants. The expressions 0.5*w*_1_*u*_1_^2^, 0.5*w*_2_*u*_2_^2^, and 0.5*w*_3_*u*_3_^2^ are costs associated with controls. The form of cost is quadratic because we assumed it to be nonlinear in nature [3]. Our goal is to minimize the population size of *W*(*t*), *I*(*t*), and *A*(*t*) through intervention with control measures *u*_1_(*t*), and *u*_2_(*t*) along with the costs associated with them. For two optimal controls *u*_1_^∗^, and *u*_2_^∗^, we have

(54) $$\begin{array}{c} J\left(u_{1}^{*},u_{2}^{*},u_{3}^{*}\right)=\min\left\{J\left(u_{1},u_{2}\right)\text{: }u_{1},u_{2}\in U\right\}, \end{array}$$

where *U* = {(*u*_1_, *u*_2_): 0 ≤ *u*_1_ ≤ 1, 0 ≤ *u*_2_ ≤ 1}, *u*_1_, *u*_2_, and *u*_3_ are measurable controls.

### 4.1. Existence of the Optimal Control

To show the existence of optimal control, we use the approach used in [3]. We have already proved that the HIV model (2) is bounded, so this result can be used to prove the existence of optimal control over finite time interval as applied in [3, 52]. To ensure the existence of optimal control, we need to check if the following conditions are satisfied:

- The set of controls and state variables be nonempty
- The control set *U* is convex and closed
- The right hand side of the state system is bounded by a linear function in the state and control variables
- The integrand of objective functional is convex on *U*
- The integrand of objective functional is bounded below by *k*_2_ − *k*_1_(|*u*_1_|^2^ + |*u*_2_|^2^)^*k*/2^.*k*_1_, *k*_2_ > 0 and *k* > 1

An existence of the state system with bounded coefficients used to give condition (i). The control set is convex and closed by definition. The right hand side of the state system satisfies (iii). The state solutions are already bounded (iv). The integrand in the objective functional *c*_1_*W* + *c*_2_*I* + *c*_3_*A* + (1/2)(*w*_1_*u*_1_^2^ + *w*_2_*u*_2_^2^) is clearly convex on *U*. (v) Further, from restriction on control measures, we have

(55) $$\begin{array}{c} \frac{1}{2}w_{i}u_{i}^{2}\leq \frac{1}{2}w_{i},u_{i}\in \left[0,1\right]. \end{array}$$

Also, considering the preceding inequality, the integrand can be written as

(56) $$\begin{array}{c} c_{1}W+c_{2}I+c_{3}A+\frac{1}{2}\left(w_{1}u_{1}^{2}+w_{2}u_{2}^{2}\right)\geq k_{1}{\left({\left|u_{1}\right|}^{2}+{\left|u_{2}\right|}^{2}\right)}^{k/2}-k_{2}. \end{array}$$

where *k*_1_ = min(*w*_1_/2, *w*_2_/2), *k*_2_ = *w*_2_/2, *k* = 2.

Therefore, there exists optimal control measures *u*_1_ and *u*_2_ that minimize the objective functional $J\left(\begin{array}{c} u_{1},u_{2}, \end{array}\right)$.

### 4.2. The Hamiltonian and Optimality System

The Pontryagin maximum principle stated the necessary conditions which are satisfied by optimal pair. Hence, by this principle, we obtained the Hamiltonian function (*H*) defined as

(57) $$\begin{array}{c} H\left(S,W,I,U,A,\right)=c_{1}W+c_{2}I+c_{3}A+\frac{1}{2}\left(w_{1}u_{1}^{2}+w_{2}u_{2}^{2}\right)+\lambda_{1}\frac{dS}{dt}+\lambda_{2}\frac{dW}{dt}+\lambda_{3}\frac{dI}{dt}+\lambda_{4}\frac{dU}{dt}+\lambda_{5}\frac{dA}{dt}, \end{array}$$

where, *λ*_*i*_, *i* = 1, 2, 3, 4, 5 are the adjoint variables corresponding to state variables *S*, *W*, *I*, *U*, and *A*, respectively, and to be determined using Pontryagin's maximal principle for the existence of optimal pairs.

**Theorem 8 .**

Let *S*, *W*, *I*, *U*, and *A* are optimal state solutions with associated optimal control variables *u*_1_, *u*_2_, and *u*_3_ for the optimal control model, there exist co-state variables *λ*_1_, *λ*_2_, *λ*_3_, *λ*_4_, and *λ*_5_ that satisfy

(58) $$\begin{array}{c} \frac{d\lambda_{1}}{dt}=-\frac{\partial H}{\partial S},\frac{d\lambda_{2}}{dt}=-\frac{\partial H}{\partial W},\frac{d\lambda_{3}}{dt}=-\frac{\partial H}{\partial I},\frac{d\lambda_{4}}{dt}=-\frac{\partial H}{\partial U},\frac{d\lambda_{5}}{dt}=-\frac{\partial H}{\partial A}. \end{array}$$

With transversality or final time conditions, *λ*_1_(*t*_*f*_) = *λ*_2_(*t*_*f*_) = *λ*_3_(*t*_*f*_) = *λ*_4_(*t*_*f*_) = *λ*_5_(*t*_*f*_) = 0 and where *H* is Hamiltonian function given in (∗). Furthermore, the optimal controls *u*_1_^∗^, *u*_2_^∗^, and *u*_3_^∗^ are

(59) $$\begin{array}{c} u_{1}^{*}=\min\left\{1,\max\left\{\frac{\beta SI\left(\lambda_{2}-\lambda_{1}\right)}{w_{1}N},0\right\}\right\},\\ u_{2}^{*}=\min\left\{1,\max\left\{\frac{\eta I\left(\lambda_{5}-\lambda_{3}\right)+kA\left(\lambda_{5}-\lambda_{3}\right)+\theta I\left(\lambda_{3}-\lambda_{4}\right)}{w_{2}},0\right\}\right\}. \end{array}$$

Over the constraints,

(60) $$\begin{array}{c} 0\leq u_{1}\leq 1,0\leq u_{2}\leq 1. \end{array}$$

**Proof**

Pontryagin's maximum principle gives the standard form of adjoint equation with transversality conditions [40]. Now, differentiating the Hamiltonian function with respect to state variables *S*, *W*, *I*, *U* and *A* , respectively, the adjoint equations can be written as

(61) $$\begin{array}{c} \frac{d\lambda_{1}}{dt}=-\frac{\partial H}{\partial S}=\left(1-u_{1}\right)\frac{\left(\beta_{1}W+\beta_{2}I\right)N-S\left(\beta_{1}W+\beta_{2}I\right)}{N^{2}}\left(\lambda_{1}-\lambda_{2}\right)+\mu \lambda_{1},\\ \frac{d\lambda_{2}}{dt}=-\frac{\partial H}{\partial W}=-c_{1}+\left(1-u_{1}\right)\frac{\beta_{1}SN-S\left(\beta_{1}W+\beta_{2}I\right)}{N^{2}}\left(\lambda_{1}-\lambda_{2}\right)+\xi \left(\lambda_{2}-\lambda_{3}\right)+\mu \lambda_{2},\\ \frac{d\lambda_{3}}{dt}=-\frac{\partial H}{\partial I}=-c_{2}+\left(1-u_{1}\right)\frac{\beta_{2}SN-S\left(\beta_{1}W+\beta_{2}I\right)}{N^{2}}\left(\lambda_{1}-\lambda_{2}\right)+\left(1-u_{2}\right)\eta \left(\lambda_{3}-\lambda_{5}\right)+u_{2}\theta \left(\lambda_{3}-\lambda_{4}\right)+\mu \lambda_{3},\\ \frac{d\lambda_{4}}{dt}=-\frac{\partial H}{\partial U}=\phi \left(\lambda_{4}-\lambda_{3}\right)+\mu \lambda_{4},\\ \frac{d\lambda_{5}}{dt}=-\frac{\partial H}{\partial A}=-c_{3}+u_{2}\kappa \left(\lambda_{5}-\lambda_{3}\right)+\left(\delta +\mu \right)\lambda_{5}. \end{array}$$

Further, the characterization of optimal controls *u*_1_^∗^, and *u*_2_^∗^ shows that

(62) $$\begin{array}{c} \frac{\partial H}{\partial u_{1}}=\frac{\partial H}{\partial u_{2}}=0. \end{array}$$

It follows that the optimal solution subject to constraints 0 ≤ *u*_1_ ≤ 1, 0 ≤ *u*_2_ ≤ 1 is

(63) $$\begin{array}{c} u_{1}^{*}=u_{1}=\frac{S\left(\beta_{1}W+\beta_{2}I\right)}{w_{1}N}\left(\lambda_{2}-\lambda_{1}\right),u_{2}^{*}=u_{2}=\frac{\eta I\left(\lambda_{5}-\lambda_{3}\right)+kA\left(\lambda_{5}-\lambda_{3}\right)+\theta I\left(\lambda_{3}-\lambda_{4}\right)}{w_{2}}. \end{array}$$

Therefore, considering the bounds of the control, the optimal control variables are given by

(64) $$\begin{array}{c} u_{1}^{*}=\left\{\begin{array}{cc} \frac{S\left(\beta_{1}W+\beta_{2}I\right)}{w_{1}N}\left(\lambda_{2}-\lambda_{1}\right), & \text{if}\;0<\frac{S\left(\beta_{1}W+\beta_{2}I\right)}{w_{1}N}\left(\lambda_{2}-\lambda_{1}\right)<1,\\ 0, & \text{if}\;\frac{S\left(\beta_{1}W+\beta_{2}I\right)}{w_{1}N}\left(\lambda_{2}-\lambda_{1}\right)\leq 0,\\ 1, & \text{if}\;1\leq \frac{S\left(\beta_{1}W+\beta_{2}I\right)}{w_{1}N}\left(\lambda_{2}-\lambda_{1}\right), \end{array}\right.\\ u_{2}^{*}=\left\{\begin{array}{cc} \frac{\eta I\left(\lambda_{5}-\lambda_{3}\right)+kA\left(\lambda_{5}-\lambda_{3}\right)+\theta I\left(\lambda_{3}-\lambda_{4}\right)}{w_{2}}, & \text{if}\;0<\frac{\eta I\left(\lambda_{5}-\lambda_{3}\right)+kA\left(\lambda_{5}-\lambda_{3}\right)+\theta I\left(\lambda_{3}-\lambda_{4}\right)}{w_{2}}<1,\\ 0, & \text{if}\;\frac{\eta I\left(\lambda_{5}-\lambda_{3}\right)+kA\left(\lambda_{5}-\lambda_{3}\right)+\theta I\left(\lambda_{3}-\lambda_{4}\right)}{w_{2}}\leq 0,\\ 1, & \text{ if}\;1\leq \frac{\eta I\left(\lambda_{5}-\lambda_{3}\right)+kA\left(\lambda_{5}-\lambda_{3}\right)+\theta I\left(\lambda_{3}-\lambda_{4}\right)}{w_{2}}. \end{array}\right. \end{array}$$

In compact form, the optimal controls can be written as

(65) $$\begin{array}{c} u_{1}^{*}=\min\left\{1,\max\left\{\frac{S\left(\beta_{1}W+\beta_{2}I\right)}{w_{1}N}\left(\lambda_{2}-\lambda_{1}\right),0\right\}\right\},\\ u_{2}^{*}=\min\left\{1,\max\left\{\frac{\eta I\left(\lambda_{5}-\lambda_{3}\right)+kA\left(\lambda_{5}-\lambda_{3}\right)+\theta I\left(\lambda_{3}-\lambda_{4}\right)}{w_{2}},0\right\}\right\}. \end{array}$$

Next, we write the optimality system using a state variables system of equations with initial conditions, a costate variables system of equations with final time conditions and optimal control solution.

(66) $$\begin{array}{c} \frac{dS}{dt}=\lambda -\left(1-u_{1}\right)\frac{S\left(\beta_{1}W+\beta_{2}I\right)}{N}-\mu S,\\ \frac{dW}{dt}=\left(1-u_{1}\right)\frac{S\left(\beta_{1}W+\beta_{2}I\right)}{N}-\left(\xi +\mu \right)W,\\ \frac{dI}{dt}=\xi W+u_{2}\kappa A+\phi U-\left(\left(1-u_{2}\right)\eta +u_{2}\theta +\mu \right)I,\\ \frac{dU}{dt}=u_{2}\theta I-\left(\emptyset +\mu \right)U,\\ \frac{dA}{dt}=\left(1-u_{2}\right)\eta I-\left(u_{2}kA+\delta +\mu \right)A,\\ \frac{d\lambda_{1}}{dt}=-\frac{\partial H}{\partial S}=\left(1-u_{1}\right)\frac{\left(\beta_{1}W+\beta_{2}I\right)N-S\left(\beta_{1}W+\beta_{2}I\right)}{N^{2}}\left(\lambda_{1}-\lambda_{2}\right)+\mu \lambda_{1},\\ \frac{d\lambda_{2}}{dt}=-\frac{\partial H}{\partial W}=-c_{1}+\left(1-u_{1}\right)\frac{\beta_{1}SN-S\left(\beta_{1}W+\beta_{2}I\right)}{N^{2}}\left(\lambda_{1}-\lambda_{2}\right)+\xi \left(\lambda_{2}-\lambda_{3}\right)+\mu \lambda_{2},\\ \frac{d\lambda_{3}}{dt}=-\frac{\partial H}{\partial I}=-c_{2}+\left(1-u_{1}\right)\frac{\beta_{2}SN-S\left(\beta_{1}W+\beta_{2}I\right)}{N^{2}}\left(\lambda_{1}-\lambda_{2}\right)+\left(1-u_{2}\right)\eta \left(\lambda_{3}-\lambda_{5}\right)+\theta \left(\lambda_{3}-\lambda_{4}\right)+\mu \lambda_{3},\\ \frac{d\lambda_{4}}{dt}=-\frac{\partial H}{\partial U}=\phi \left(\lambda_{4}-\lambda_{3}\right)+\mu \lambda_{4},\\ \frac{d\lambda_{5}}{dt}=-\frac{\partial H}{\partial A}=-c_{3}+u_{3}\kappa \left(\lambda_{5}-\lambda_{3}\right)+\left(\delta +\mu \right)\lambda_{5}, \end{array}$$

with conditions *λ*_1_(*t*_*f*_) = *λ*_2_(*t*_*f*_) = *λ*_3_(*t*_*f*_) = *λ*_4_(*t*_*f*_) = *λ*_5_(*t*_*f*_) = 0, *S*(0) = *S*_0_, *W*(0) = *W*_0_, *I*(0) = *I*_0_, *U*(0) = *U*_0_, and *A*(0) = *A*_0_.

## 5. Numerical Simulations

Next, we investigate qualitatively the effect of optimal control strategies on the spread of HIV in a population. Hence, we categorized strategies as follows:

- Strategy 1: only prevention control effort (*u*_1_)
- Strategy 2: only treatment control effort (*u*_2_)
- Strategy 3: both treatment and prevention control efforts (*u*_1_ and *u*_2_)

Solving the optimality system yields the better control. For solving the optimality system, an iterative scheme is used. We begin by using the fourth order Runge–Kutta scheme to solve the state equations with a guess for the controls over simulated time. The adjoint equations are solved using the current iterations solutions of the state equation using the backward fourth-order Runge–Kutta scheme because of the transversality conditions. The controls are then updated by combining the previous controls with the value from the characterizations. In this section, numerical simulations are done to illustrate the analytical results obtained in the above analysis. The initial values for variables of model (2) are *S*(0) = 6000, *W*(0) = 200, *I*(0) = 1000, *U*(0) = 300, *A*(0) = 100, *c*_1_ = 20, *c*_2_ = 15, *c*_3_ = 25, *w*_1_ = 1, and *w*_2_ = 1. Moreover, the value and the source of parameters used in the simulations are given in the Table 4.

**Figure 2:**
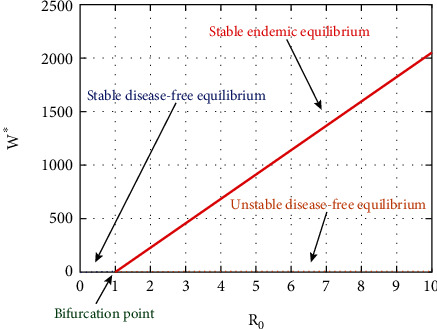
Forward bifurcation diagram of model (2).

In Figure 2, the endemic equilibrium changes its stability from unstable to stable and disease-free equilibrium changes its stability from stable to unstable at the bifurcation point *R*_0_ = 1. Moreover, the point *R*_0_ = 1 is the critical point where forward bifurcation behavior of model is exhibited. Epidemiologically, interpretation gives the disease persists in the population if *R*_0_ > 1 and extinct if *R*_0_ ≤ 1

**Figure 3:**
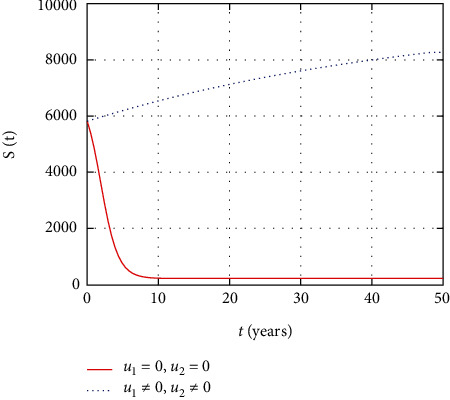
Comparison of susceptible population without and with control.

In Figure 3, the susceptible population dynamics is simulated considering the prevention and treatment control interventions. The susceptible population size increases due to intervention with prevention control. However, without control, more people get infected and the size of the susceptible population decreases.

**Figure 4:**
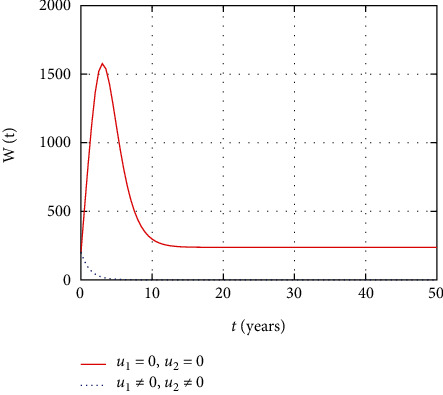
Comparison of asymptomatic population without and with control.

In Figure 4, the asymptomatic population dynamics is simulated considering the prevention and treatment control interventions. The asymptomatic population size decrease due to intervention with prevention control. However, without control, more people get infected and the size of the asymptomatic population increases with time.

**Figure 5:**
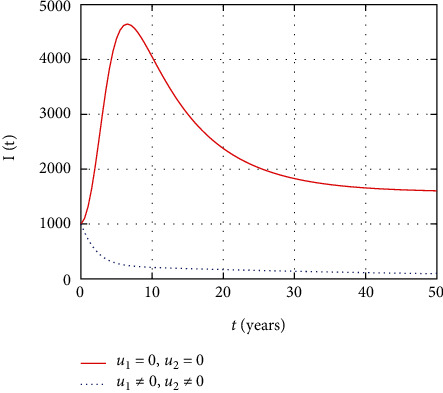
Comparison of symptomatic HIV population without and with control.

In Figure 5, the symptomatic HIV population dynamics is simulated considering the prevention and treatment control interventions. The symptomatic HIV population size decreases due to intervention with control measures. However, without control, more people get infected and the size of the HIV population increases with time.

**Figure 6:**
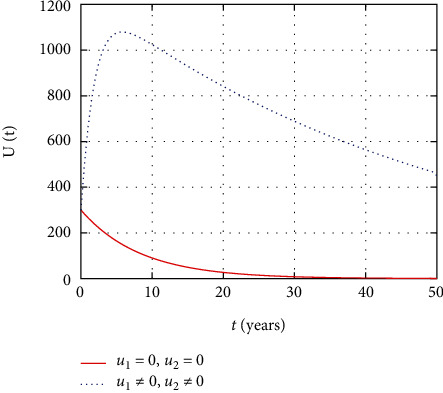
Comparison of susceptible population without and with control.

In Figure 6, the undetectable population dynamics is simulated considering the prevention and treatment control interventions. The undetectable population size decreases without intervention with controls due to replication of virus without control. However, with effective adherence to controls, the size of the undetectable population increases with time.

**Figure 7:**
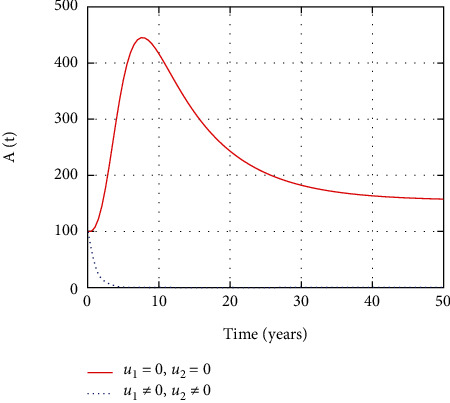
Comparison of AIDS population without and with control.

In Figure 7, the AIDS population dynamics is simulated considering the prevention and treatment control interventions. The AIDS population size decreases due to intervention with controls. However, without controls, more people severely attacked with HIV and progress to advanced stage so that the size of the AIDS population increases.

**Figure 8:**
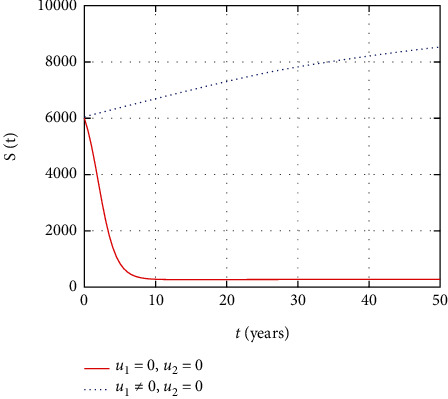
Comparison of susceptible population without treatment.

In Figure 8, the susceptible population dynamics is simulated considering only the prevention control. The susceptible population size increase due to intervention with prevention control. However, without prevention control, people get infected and the size of the susceptible population decreases with time.

**Figure 9:**
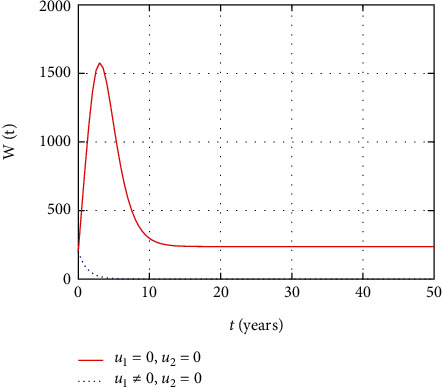
Comparison of asymptomatic population without treatment.

In Figure 9, the asymptomatic population dynamics is simulated considering the prevention control intervention. The asymptomatic population size decreases due to intervention with prevention control as less number of individuals get infected in the presence of prevention control. However, without control, more people get infected and the size of the asymptomatic population increases with time.

**Figure 10:**
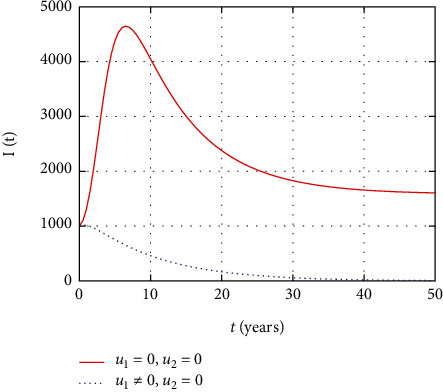
Comparison of HIV population without treatment.

In Figure 10, the symptomatic HIV population dynamics is simulated considering the prevention control intervention. The symptomatic HIV population size increases without intervention with prevention control. However, the prevention control reduces the number of individuals infected whose impact reduces the number of symptomatic HIV population.

**Figure 11:**
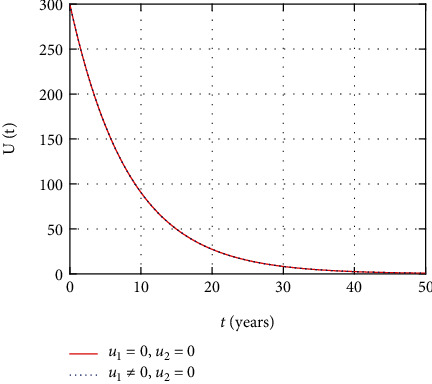
Comparison of undetectable population without treatment.

In Figure 11, the undetectable population dynamics is simulated considering the only prevention control intervention. The undetectable population size remains the same without or with presence of prevention control due to absence of treatment control intervention.

**Figure 12:**
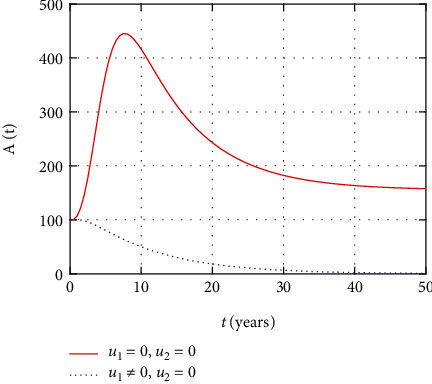
Comparison of AIDS population without treatment.

In Figure 12, the AIDS population dynamics is simulated considering the prevention control intervention. The AIDS population size increases without treatment control intervention control. However, with control, less people progress to the advanced stage of HIV and the size of the AIDS population decreases with time.

**Figure 13:**
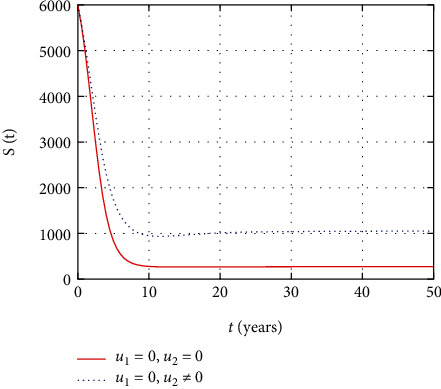
Comparison of susceptible population without prevention control.

In Figure 13, the susceptible population dynamics is simulated considering only treatment control intervention. The HIV population size increases with treatment control intervention control. However, without treatment control, more people get infected and the size of susceptible population decreases.

**Figure 14:**
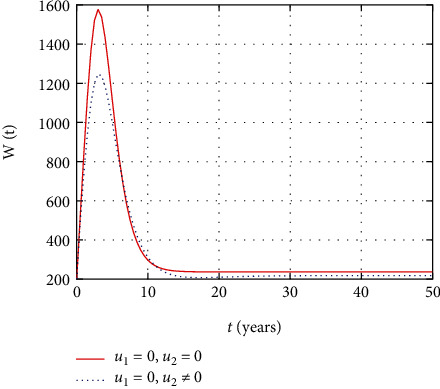
Comparison of asymptomatic population without prevention control.

Figure 14 shows that the absence of the prevention control measure increases the number of HIV-infected individuals in the asymptomatic stage whereas the intervention with only treatment reduces the number HIV-infected individuals in the asymptomatic stage.

**Figure 15:**
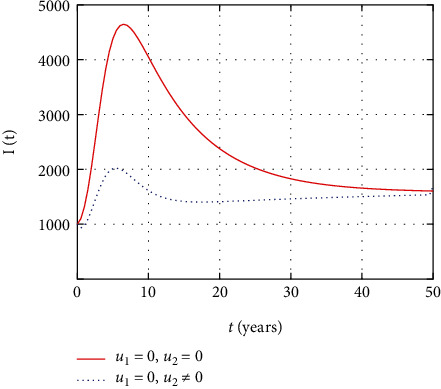
Comparison of HIV population without prevention control.

In Figure 15, the symptomatic HIV-infected individuals increase the HIV population without control measures, but the early intervention with treatment reduces the number of individuals that get serious with virus.

**Figure 16:**
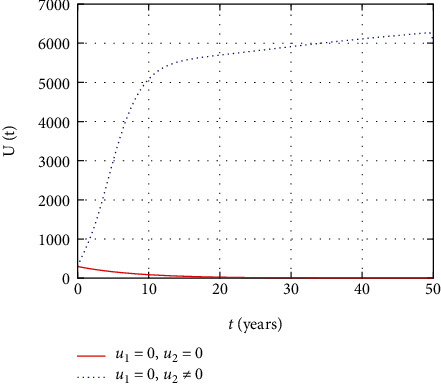
Comparison of undetectable population without prevention control.

In Figure 16, the dynamics of undetectable stage HIV-infected individuals are described. The simulation shows that the number of undetectable individuals increases continuously with continuous using of treatment, but in the absence or stopping the medicine causes the number of individuals at the undetectable individuals to become detectable.

**Figure 17:**
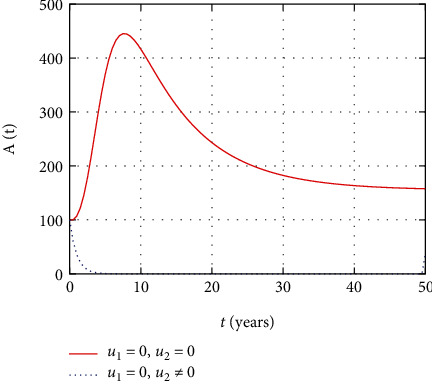
Comparison of AIDS population without prevention control.

Figure 17 shows that the number of individuals at AIDS stage increases with time in the absence of the medicine that inhibits the replication of the viruses in the body and prevention control measures. However, early intervention with treatment reduces the number of individuals at the AIDS stage effectively.

**Figure 18:**
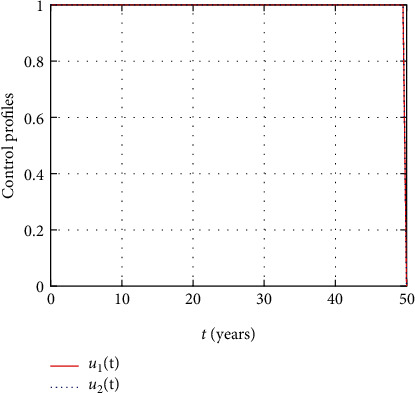
Simulation of prevention and treatment control profiles.

In Figure 18, the prevention and the treatment control profiles are simulated. The simulation results indicate that early and continuous application of the prevention and treatment control measures is effective in the controlling the HIV infection.

**Figure 19:**
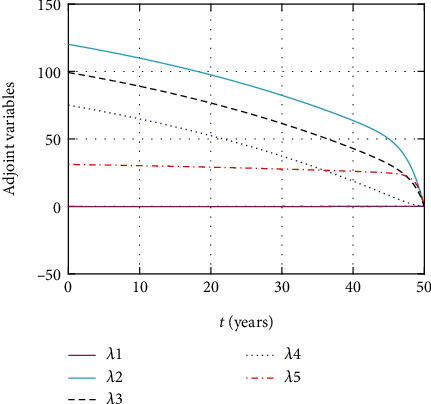
Simulation of adjoint variables for the given time interval.

In Figure 19, the simulation of adjoint variables are performed to show the conditions required in the analysis of optimal control problems. Moreover, at the final time, the adjoint variables yield zero values.

**Figure 20:**
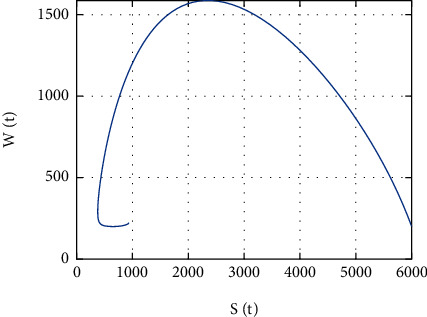
Simulation of *S*(*t*) and *W*(*t*) dynamics.

In Figure 20, the simulation of *S*(*t*) versus *W*(*t*) is performed; the size of *S*(*t*) decreases to 2000 as the size of *W*(*t*) increases to 1600. However, for size of *S*(*t*) ≤ 2000 and *W*(*t*) ≤ 1600, both population sizes increase (decrease) together.

**Figure 21:**
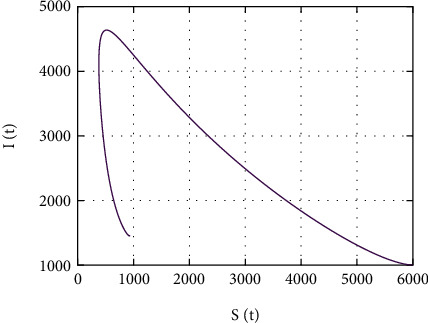
Simulation of *S*(*t*) and *I*(*t*) dynamics.

In Figure 21, the simulation of *S*(*t*) versus *I*(*t*) is performed. Initially, the size of *S*(*t*) decrease approximately to 300 as the size of *W*(*t*) increase to 4700; however, for size of *S*(*t*) ≤ 300 and *W*(*t*) ≤ 4700, both population sizes increase (decrease) together.

**Figure 22:**
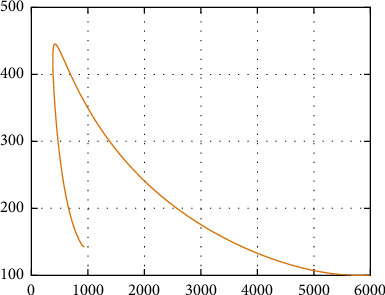
Simulation of *S*(*t*) and *I*(*t*) dynamics.

In Figure 22, the simulation of *S*(*t*) versus *A*(*t*) is performed. Initially, the size of *S*(*t*) decrease approximately to 300 as the size of *A*(*t*) increase to 430; however, for size of *S*(*t*) ≤ 300 and *A*(*t*) ≤ 430 both population sizes increase (decrease) together.

**Figure 23:**
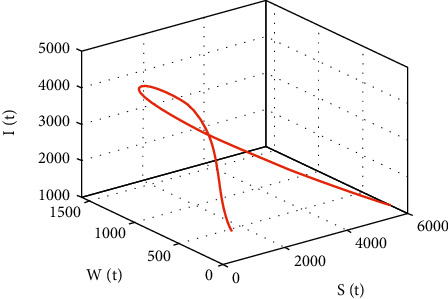
Simulation of *S*(*t*) and *I*(*t*) dynamics.

In Figure 23, the phase portrait of *S*(*t*), *W*(*t*), and *I*(*t*) is performed. The simulation shows the initial occurrence of HIV prevalence increases. However, as time increases, less number of individuals becomes susceptible with the presence of HIV-infected individuals.

### 5.1. Cost-Effectiveness Analysis

Incremental cost-effectiveness ratio (ICER) used to compare the differences between the costs and health outcomes of two alternative intervention strategies that compete for the same resources and is generally described as the additional cost per additional health outcome [54, 55].

(67) $$\begin{array}{c} \text{ICER}=\frac{\text{Differences between the costs }}{\text{Difference between health outcomes }}. \end{array}$$

In ICER, when comparing two competing intervention strategies incrementally, one intervention should be compared with the next less effective alternative.

The ICER is computed as follows:

(68) $$\begin{array}{c} \text{ICER }\left(2\right)=\frac{32187}{115950}=0.2776,\\ \text{ICER }\left(1\right)=\frac{6437.4-32187}{274020-115950}=-0.1629. \end{array}$$

Comparing the computations, the ICER (1) < *I*CER (2). This implies to discard strategy 2 and construct a table to compare strategies 1 and 3.

The ICER is computed as follows:

(69) $$\begin{array}{c} \text{ICER}\left(1\right)=\frac{\;6437.4}{274020}=0.0235,\\ \text{ICER}\left(3\right)=\frac{\;38624-6437.4}{281350-274020}=4.3911. \end{array}$$

Comparing the computations, the ICER (1) < ICER (3). This implies that strategy (2) is more effective and economical over strategy (6). Therefore, discard strategy 3 and select strategy 1 as a better intervention for optimal control of HIV transmission dynamics. Therefore, based on the results obtained from Tables 5–7, the prevention control strategy is more cost-effective strategy than other control strategies to overcome the burden of HIV on human life.

### 5.2. Result and Discussion

In this section, we present the result and discuss the results obtained from analytical analysis and numerical simulations of model (2). Particularly, we discuss the impact of ART on HIV transmission as a result of varying the value of transfer rate parameter. In Figure 1, the dynamics of population is described by schematic diagram and the model is developed using it. In Figure 2, we observe that the endemic equilibrium exhibits unstable state behavior if *R*_0_ < 1. But, it exhibits global asymptotic stable state behavior if *R*_0_ > 1. On the other hand, DFE is stable for *R*_0_ < 1 and unstable if *R*_0_ > 1. Moreover, it can be observed that the system shifts from the stable DFE to unstable at the bifurcation point *R*_0_ = 1. That is, there is forward bifurcation at the critical point. Further, in Figures 3–17, the impact of optimal controls is described clearly. Moreover, the presence of optimal control measures increases the number of susceptible individuals. However, the presence of optimal control measures decreases the number of individuals in the *W*, *I*, and *A* classes. In Figure 18, the prevention and the treatment control profiles are simulated. The simulations results indicate early and continuous application of the prevention and treatment control measures are effective in the controlling the HIV infection, whereas in Figure 19 the simulation of adjoint variables are performed to show the conditions required in the analysis of optimal control problems. Moreover, at the final time, the adjoint variables yield zero values.

Further, in Figure 20, the simulation of *S*(*t*) versus *W*(*t*) is performed; the size of *S*(*t*) decreases to 2000 as the size of *W*(*t*) increases to 1600. However, for size of *S*(*t*) ≤ 2000 and *W*(*t*) ≤ 1600, both population sizes increase (decrease) together, and in Figure 21, the simulation of *S*(*t*) versus *I*(*t*) is performed. Initially, the size of *S*(*t*) decreases approximately to 300 as the size of *W*(*t*) increases to 4700; however, for size of *S*(*t*) ≤ 300 and *W*(*t*) ≤ 4700, both population sizes increase (decrease) together; in Figure 22, the simulation of *S*(*t*) versus *A*(*t*) is performed. Initially, the size of *S*(*t*) decreases approximately to 300 as the size of *A*(*t*) increases to 430; however, for size of *S*(*t*) ≤ 300 and *A*(*t*) ≤ 430, both population sizes increase (decrease) together. In Figure 23, the phase portrait of *S*(*t*), *W*(*t*), and *I*(*t*) is performed. The simulation shows the initial occurrence of HIV prevalence increases. However, as time increases, less number of individuals becomes susceptible with presence of HIV-infected individuals.

Also, the investigation of cost-effectiveness analysis with possible combination with prevention and treatment control measures for HIV infection shows that early applying of prevention control measures is a better strategy than applying only ART or combined strategy with prevention and treatment control measures.

### 5.3. Conclusion

In this study, we have modified the HIV model by including an undetectable compartment that emphasizes the impact of using ART properly and continuously in the entire period of HIV infection. Also, the study identified the impact of faulty using ART by varying the parameter values that control right and faulty of using ART. From sensitivity analysis, it is concluded that increasing proper and continuous use of ART help significantly to reduce the number of reproduction number. Moreover, the analytical and numerical simulation results show that the disease-free equilibrium is both locally and globally stable for *R*_0_ ≤ 1. But, the endemic equilibrium is both locally and globally stable for *R*_0_ > 1. Furthermore, at the threshold point *R*_0_ = 1, the system experiences a forward branch. Analysis results show that HIV infection can be controlled with proper administration and use of ART. Particularly, with prevention and treatment as the optimal control measures, it reduces the number of individuals living with HIV and the progression of the HIV patient to the AIDS stage. Also, examining cost-benefit analyses of possible combinations of preventive and therapeutic management measures for HIV infection shows that preventive control measure is a better strategy if applied earlier effectively than using ART alone or combined.

## Figures and Tables

**Table 1:** Notations and description of model variables.

Variable	Description
*S*(*t*)	Size of susceptible population at time *t*
*W*(*t*)	Size of HIV untested population at time *t*
*I*(*t*)	Size of HIV tested pre-AIDS population with transmittable virus at time *t*.
*U*(*t*)	Size of pre-AIDS population with untransmittable virus at time *t*
*A*(*t*)	Size of AIDS population at time *t*

**Table 2:** Model parameter notations and description.

Parameter	Description
*β*	Transmission rate of HIV to susceptible population
*λ*	Recruitment rate of individuals to susceptible class
*θ*	Transferring rate of population from compartment *I* to *U* as a result of using ART properly
*ϕ*	Progression rate of population from compartment *U* to *I* as a result of faulty using ART.
*ξ*	Progression rate of population from compartment *W* to *I*
*α*	Progression rate of *I* to *A*
*κ*	Transferring rate of *I* to *A* due to treatment
*μ*	Natural death rate of all population
*δ*	Disease induced death rate of AIDS population

**Table 3:** Sensitivity index value of *R*_0_ with respect parameter.

Parameter	Value	Sensitivity index value	Description
*β*_1_	0.9815	+0.145	*β*_1_ ~ *R*_0_
*β*_2_	0.866	+0.854	*β*_2_ ~ *R*_0_
*ξ*	0.8	-0.121	$\xi \sim \frac{1}{R_{0}}\;$
*μ*	0.02	-0.004	$\mu \sim \frac{1}{R_{0}}$
*η*	0.1	-0.701	$\eta \sim \frac{1}{R_{0}}$

**Table 4:** Value and source of parameters used in the simulation.

Parameter	Value	Source
*λ*	200/year	Assumed
*β*_1_	0.9815/year	Assumed
*β*_2_	0.866/year	[9]
*μ*	0.02/year	[36]
*ξ*	0.8/year	Assumed
*η*	0.1 /year	[7]
*ϕ*	0.1/year	[7]
*θ*	0.5/year	Assumed
*κ*	0.1/year	[30]
*δ*	1/year	[30]

**Table 5:** Rank of strategies based on the number of the infections averted.

Strategies	Total infections averted	Total costs	ICER
No strategy	0	0	—
Strategy 1 (prevention)	274020	32187	0
Strategy 2 (treatment)	115950	6437.4	0
Strategy 3 (prevention and treatment)	281350	2574.9	0

**Table 6:** Comparison between strategies 2 and 1.

Strategies	Total averted	Total costs	ICER
No strategy	0	0	—
Strategy 2 (treatment)	115950	32187	0.2776
Strategy 1 (prevention)	274020	6437.4	−0.1629

**Table 7:** Comparison between strategies 1 and 3.

Strategies	Total averted	Total costs	ICER
No strategy	0	0	—
Strategy 1 (prevention)	274020	6437.4	0.0235
Strategy 3 (prevention and treatment)	281350	38624	4.3911

## Data Availability

All data used in the analysis of the model are included in the manuscript.

## Appendix

### A. Partial Derivatives of Functions Used in the Bifurcation Analysis

(A.1)
$$\begin{array}{c} \frac{\partial^{2}f_{1}}{\partial x_{1}^{2}}=\frac{2\left(\beta_{1}x_{2}+\beta_{2}x_{3}\right)}{{\left(x_{1}+x_{2}+x_{3}+x_{4}+x_{5}\right)}^{2}}-\frac{2\left(\beta_{1}x_{1}x_{2}+\beta_{2}x_{1}x_{3}\right)}{{\left(x_{1}+x_{2}+x_{3}+x_{4}+x_{5}\right)}^{3}},\\ \frac{\partial^{2}f_{1}}{\partial x_{1}\partial x_{2}}=-\frac{\beta_{1}}{x_{1}+x_{2}+x_{3}+x_{4}+x_{5}}+\frac{\beta_{1}x_{1}}{{\left(x_{1}+x_{2}+x_{3}+x_{4}+x_{5}\right)}^{2}}+\frac{\beta_{1}x_{2}+\beta_{2}x_{3}}{{\left(x_{1}+x_{2}+x_{3}+x_{4}+x_{5}\right)}^{2}}-\frac{2\left(\beta_{1}x_{1}x_{2}+\beta_{2}x_{1}x_{3}\right)}{{\left(x_{1}+x_{2}+x_{3}+x_{4}+x_{5}\right)}^{3}}=\frac{\partial^{2}f_{1}}{\partial x_{2}\partial x_{1}},\\ \frac{\partial^{2}f_{1}}{\partial x_{2}^{2}}=\frac{2\beta_{1}x_{1}}{{\left(x_{1}+x_{2}+x_{3}+x_{4}+x_{5}\right)}^{2}}-\frac{2\left(\beta_{1}x_{1}x_{2}+\beta_{2}x_{1}x_{3}\right)}{{\left(x_{1}+x_{2}+x_{3}+x_{4}+x_{5}\right)}^{3}},\\ \frac{\partial^{2}f_{1}}{\partial x_{3}\partial x_{2}}=\frac{\beta_{1}x_{1}}{{\left(x_{1}+x_{2}+x_{3}+x_{4}+x_{5}\right)}^{2}}+\frac{\beta_{2}x_{1}}{{\left(x_{1}+x_{2}+x_{3}+x_{4}+x_{5}\right)}^{2}}-\frac{2\left(\beta_{1}x_{1}x_{2}+\beta_{2}x_{1}x_{3}\right)}{{\left(x_{1}+x_{2}+x_{3}+x_{4}+x_{5}\right)}^{3}}=\frac{\partial^{2}f_{1}}{\partial x_{2}\partial x_{3}},\\ \frac{\partial^{2}f_{1}}{\;\partial x_{4}\partial x_{2}}=\frac{\beta_{1}x_{1}}{{\left(x_{1}+x_{2}+x_{3}+x_{4}+x_{5}\right)}^{2}}-\frac{2\left(\beta_{1}x_{1}x_{2}+\beta_{2}x_{1}x_{3}\right)}{{\left(x_{1}+x_{2}+x_{3}+x_{4}+x_{5}\right)}^{3}}=\frac{\partial^{2}f_{1}}{\partial x_{2}\partial x_{4}},\\ \frac{\partial^{2}f_{1}}{\partial x_{3}^{2}}=\frac{2\beta_{2}x_{1}}{{\left(x_{1}+x_{2}+x_{3}+x_{4}+x_{5}\right)}^{2}}-\frac{2\left(\beta_{1}x_{1}x_{2}+\beta_{2}x_{1}x_{3}\right)}{{\left(x_{1}+x_{2}+x_{3}+x_{4}+x_{5}\right)}^{3}},\\ \frac{\partial^{2}f_{1}}{\partial x_{1}\partial x_{3}}=-\frac{\beta_{2}}{x_{1}+x_{2}+x_{3}+x_{4}+x_{5}}+\frac{\beta_{2}x_{1}}{{\left(x_{1}+x_{2}+x_{3}+x_{4}+x_{5}\right)}^{2}}+\frac{\beta_{1}x_{2}+\beta_{2}x_{3}}{{\left(x_{1}+x_{2}+x_{3}+x_{4}+x_{5}\right)}^{2}}-\frac{2\left(\beta_{1}x_{1}x_{2}+\beta_{2}x_{1}x_{3}\right)}{{\left(x_{1}+x_{2}+x_{3}+x_{4}+x_{5}\right)}^{3}}=\frac{\partial^{2}f_{1}}{\partial x_{3}\partial x_{1}},\\ \frac{\partial^{2}f_{1}}{\partial x_{4}\partial x_{3}}=\frac{\beta_{2}x_{1}}{{\left(x_{1}+x_{2}+x_{3}+x_{4}\right)}^{2}}-\frac{2\left(\beta_{1}x_{1}x_{2}+\beta_{2}x_{1}x_{3}\right)}{{\left(x_{1}+x_{2}+x_{3}+x_{4}\right)}^{3}}=\frac{\partial^{2}f_{1}}{\partial x_{3}\partial x_{4}},\\ \frac{\partial^{2}f_{1}}{\partial x_{4}^{2}}=-\frac{2\left(\beta_{1}x_{1}x_{2}+\beta_{2}x_{1}x_{3}\right)}{{\left(x_{1}+x_{2}+x_{3}+x_{4}\right)}^{3}},\\ \frac{\partial^{2}f_{1}}{\partial x_{1}\partial x_{4}}=\frac{\beta_{1}x_{2}+\beta_{2}x_{3}}{{\left(x_{1}+x_{2}+x_{3}+x_{4}\right)}^{2}}-\frac{2\left(\beta_{1}x_{1}x_{2}+\beta_{2}x_{1}x_{3}\right)}{{\left(x_{1}+x_{2}+x_{3}+x_{4}\right)}^{3}}=\frac{\partial^{2}f_{2}}{\partial x_{4}\partial x_{1}},\\ \frac{\partial^{2}f_{2}}{\partial x_{1}^{2}}=-\frac{2\left(\beta_{1}x_{2}+\beta_{2}x_{3}\right)}{{\left(x_{1}+x_{2}+x_{3}+x_{4}\right)}^{2}}+\frac{2\left(\beta_{1}x_{1}x_{2}+\beta_{2}x_{1}x_{3}\right)}{{\left(x_{1}+x_{2}+x_{3}+x_{4}\right)}^{3}},\\ \frac{\partial^{2}f_{2}}{\partial x_{1}\partial x_{2}}=\frac{\beta_{1}}{x_{1}+x_{2}+x_{3}+x_{4}}-\frac{\beta_{1}x_{1}}{{\left(x_{1}+x_{2}+x_{3}+x_{4}\right)}^{2}}-\frac{\beta_{1}x_{2}+\beta_{2}x_{3}}{{\left(x_{1}+x_{2}+x_{3}+x_{4}\right)}^{2}}+\frac{2\left(\beta_{1}x_{1}x_{2}+\beta_{2}x_{1}x_{3}\right)}{{\left(x_{1}+x_{2}+x_{3}+x_{4}\right)}^{3}}=\frac{\partial^{2}f_{2}}{\partial x_{2}\partial x_{1}},\\ \frac{\partial^{2}f_{2}}{\partial x_{2}^{2}}=-\frac{2\beta_{1}x_{1}}{{\left(x_{1}+x_{2}+x_{3}+x_{4}\right)}^{2}}+\frac{2\left(\beta_{1}x_{1}x_{2}+\beta_{2}x_{1}x_{3}\right)}{{\left(x_{1}+x_{2}+x_{3}+x_{4}\right)}^{3}},\\ \frac{\partial^{2}f_{2}}{\partial x_{3}\partial x_{2}}=-\frac{\beta_{1}x_{1}}{{\left(x_{1}+x_{2}+x_{3}+x_{4}\right)}^{2}}-\frac{\beta_{2}x_{1}}{{\left(x_{1}+x_{2}+x_{3}+x_{4}\right)}^{2}}+\frac{2\left(\beta_{1}x_{1}x_{2}+\beta_{2}x_{1}x_{3}\right)}{{\left(x_{1}+x_{2}+x_{3}+x_{4}\right)}^{3}}=\frac{\partial^{2}f_{2}}{\partial x_{2}\partial x_{3}},\\ \frac{\partial^{2}f_{2}}{\partial x_{4}\partial x_{2}}=-\frac{\beta_{1}x_{1}}{{\left(x_{1}+x_{2}+x_{3}+x_{4}\right)}^{2}}+\frac{2\left(\beta_{1}x_{1}x_{2}+\beta_{2}x_{1}x_{3}\right)}{{\left(x_{1}+x_{2}+x_{3}+x_{4}\right)}^{3}}=\frac{\partial^{2}f_{2}}{\partial x_{2}\partial x_{4}},\\ \frac{\partial^{2}f_{2}}{\partial x_{3}^{2}}=-\frac{2\beta_{2}x_{1}}{{\left(x_{1}+x_{2}+x_{3}+x_{4}\right)}^{2}}+\frac{2\left(\beta_{1}x_{1}x_{2}+\beta_{2}x_{1}x_{3}\right)}{{\left(x_{1}+x_{2}+x_{3}+x_{4}\right)}^{3}},\\ \frac{\partial^{2}f_{2}}{\partial x_{1}\partial x_{3}}=\frac{\beta_{2}}{x_{1}+x_{2}+x_{3}+x_{4}}-\frac{\beta_{2}x_{1}}{{\left(x_{1}+x_{2}+x_{3}+x_{4}\right)}^{2}}-\frac{\beta_{1}x_{2}+\beta_{2}x_{3}}{{\left(x_{1}+x_{2}+x_{3}+x_{4}\right)}^{2}}+\frac{2\left(\beta_{1}x_{1}x_{2}+\beta_{2}x_{1}x_{3}\right)}{{\left(x_{1}+x_{2}+x_{3}+x_{4}\right)}^{3}}=\frac{\partial^{2}f_{2}}{\partial x_{3}\partial x_{1}},\\ \frac{\partial^{2}f_{2}}{\partial x_{4}\partial x_{3}}=-\frac{\beta_{2}x_{1}}{{\left(x_{1}+x_{2}+x_{3}+x_{4}\right)}^{2}}+\frac{2\left(\beta_{1}x_{1}x_{2}+\beta_{2}x_{1}x_{3}\right)}{{\left(x_{1}+x_{2}+x_{3}+x_{4}\right)}^{3}}=\frac{\partial^{2}f_{2}}{\partial x_{3}\partial x_{4}},\\ \frac{\partial^{2}f_{2}}{\partial x_{4}^{2}}=\frac{2\left(\beta_{1}x_{1}x_{2}+\beta_{2}x_{1}x_{3}\right)}{{\left(x_{1}+x_{2}+x_{3}+x_{4}\right)}^{3}},\\ \frac{\partial^{2}f_{2}}{\partial x_{1}\partial x_{4}}=-\frac{\beta_{1}x_{2}+\beta_{2}x_{3}}{{\left(x_{1}+x_{2}+x_{3}+x_{4}\right)}^{2}}+\frac{2\left(\beta_{1}x_{1}x_{2}+\beta_{2}x_{1}x_{3}\right)}{{\left(x_{1}+x_{2}+x_{3}+x_{4}\right)}^{3}}=\frac{\partial^{2}f_{2}}{\partial x_{4}\partial x_{1}},\\ \frac{\partial^{2}f_{3}}{\partial x_{1}^{2}}=0,\frac{\partial^{2}f_{3}}{\partial x_{1}\partial x_{2}}=0,\frac{\partial^{2}f_{3}}{\partial x_{2}^{2}}=0,\frac{\partial^{2}3}{\partial x_{3}\partial x_{2}}=0,\frac{\partial^{2}f_{3}}{\partial x_{4}\partial x_{2}}=0,\frac{\partial^{2}f_{3}}{\partial x_{3}^{2}}=0,\frac{\partial^{2}f_{3}}{\partial x_{1}\partial x_{3}}=0,\frac{\partial^{2}f_{3}}{\partial x_{4}\partial x_{3}}=0,\frac{\partial^{2}f_{3}}{\partial x_{4}^{2}}=0,\frac{\partial^{2}f_{3}}{\partial x_{1}\partial x_{4}}=0,\frac{\partial^{2}f_{4}}{\partial x_{1}^{2}}=0,\frac{\partial^{2}f_{4}}{\partial x_{1}\partial x_{2}}=0,\frac{\partial^{2}f_{4}}{\partial x_{2}^{2}}=0,\frac{\partial^{2}f_{4}}{\partial x_{3}\partial x_{2}}=0,\frac{\partial^{2}f_{4}}{\partial x_{4}\partial x_{2}}=0,\frac{\partial^{2}f_{4}}{\partial x_{3}^{2}}=0,\frac{\partial^{2}f_{4}}{\partial x_{1}\partial x_{3}}=0,\frac{\partial^{2}f_{4}}{\partial x_{4}\partial x_{3}}=0,\frac{\partial^{2}f_{4}}{\partial x_{4}^{2}}=0,\frac{\partial^{2}f_{4}}{\partial x_{1}\partial x_{4}}=0,\frac{\partial^{2}f_{5}}{\partial x_{1}^{2}}=0,\frac{\partial^{2}f_{5}}{\partial x_{1}\partial x_{2}}=0,\frac{\partial^{2}f_{5}}{\partial x_{2}^{2}}=0,\frac{\partial^{2}f_{5}}{\partial x_{3}\partial x_{2}}=0,\frac{\partial^{2}f_{5}}{\partial x_{4}\partial x_{2}}=0,\frac{\partial^{2}f_{5}}{\partial x_{3}^{2}}=0,\frac{\partial^{2}f_{5}}{\partial x_{1}\partial x_{3}}=0,\frac{\partial^{2}f_{5}}{\partial x_{4}\partial x_{3}}=0,\frac{\partial^{2}f_{5}}{\partial x_{4}^{2}}=0,\frac{\partial^{2}f_{5}}{\partial x_{1}\partial x_{4}}=0. \end{array}$$

Next, evaluating all computed second-order partial derivatives at a point (*E*_0_, *β*_1_^∗^), we obtain

(A.2) $$\begin{array}{c} \frac{\partial^{2}f_{1}}{\partial x_{2}^{2}}=\frac{2\beta_{1}}{x_{1}},\frac{\partial^{2}f_{1}}{\partial x_{3}\partial x_{2}}=\frac{\beta_{1}}{x_{1}}+\frac{\beta_{2}}{x_{1}},\frac{\partial^{2}f_{1}}{\partial x_{4}\partial x_{2}}=\frac{\beta_{1}}{x_{1}},\frac{\partial^{2}f_{1}}{\partial x_{3}^{2}}=\frac{2\beta_{2}}{x_{1}},\frac{\partial^{2}f_{1}}{\partial x_{4}\partial x_{3}}=\frac{\beta_{2}}{x_{1}},\frac{\partial^{2}f_{2}}{\partial x_{2}^{2}}=-\frac{2\beta_{1}}{x_{1}},\frac{\partial^{2}f_{2}}{\partial x_{3}\partial x_{2}}=-\frac{\beta_{1}}{x_{1}}-\frac{\beta_{2}}{x_{1}},\frac{\partial^{2}f_{2}}{\partial x_{4}\partial x_{2}}=-\frac{\beta_{1}}{x_{1}},\frac{\partial^{2}f_{2}}{\partial x_{3}^{2}}=-\frac{2\beta_{2}}{x_{1}},\frac{\partial^{2}f_{2}}{\partial x_{4}\partial x_{3}}=-\frac{\beta_{2}}{x_{1}},\frac{\partial^{2}f_{2}}{\partial x_{1}\partial x_{3}}=0,\frac{\partial^{2}f_{1}}{\partial x_{1}^{2}}=0,\frac{\partial^{2}f_{1}}{\partial x_{1}\partial x_{2}}=0,\frac{\partial^{2}f_{1}}{\partial x_{1}\partial x_{3}}=0,\frac{\partial^{2}f_{1}}{\partial x_{4}^{2}}=0,\frac{\partial^{2}f_{1}}{\partial x_{1}\partial x_{4}}=0,\frac{\partial^{2}f_{2}}{\partial x_{1}^{2}}=0,\frac{\partial^{2}f_{2}}{\partial x_{1}\partial x_{2}}=0,\frac{\partial^{2}f_{2}}{\partial x_{4}^{2}}=0,\frac{\partial^{2}f_{2}}{\partial x_{1}\partial x_{4}}=0,\frac{\partial^{2}f_{3}}{\partial x_{1}^{2}}=0,\frac{\partial^{2}f_{3}}{\partial x_{1}\partial x_{2}}=0,\frac{\partial^{2}f_{3}}{\partial x_{2}^{2}}=0,\frac{\partial^{2}3}{\partial x_{3}\partial x_{2}}=0,\frac{\partial^{2}f_{3}}{\partial x_{4}\partial x_{2}}=0,\frac{\partial^{2}f_{3}}{\partial x_{3}^{2}}=0,\frac{\partial^{2}f_{3}}{\partial x_{1}\partial x_{3}}=0,\frac{\partial^{2}f_{3}}{\partial x_{4}\partial x_{3}}=0,\frac{\partial^{2}f_{3}}{\partial x_{4}^{2}}=0,\frac{\partial^{2}f_{3}}{\partial x_{1}\partial x_{4}}=0,\frac{\partial^{2}f_{4}}{\partial x_{1}^{2}}=0,\frac{\partial^{2}f_{4}}{\partial x_{1}\partial x_{2}}=0,\frac{\partial^{2}f_{4}}{\partial x_{2}^{2}}=0,\frac{\partial^{2}f_{4}}{\partial x_{3}\partial x_{2}}=0,\frac{\partial^{2}f_{4}}{\partial x_{4}\partial x_{2}}=0,\frac{\partial^{2}f_{4}}{\partial x_{3}^{2}}=0,\frac{\partial^{2}f_{4}}{\partial x_{1}\partial x_{3}}=0,\frac{\partial^{2}f_{4}}{\partial x_{4}\partial x_{3}}=0,\frac{\partial^{2}f_{4}}{\partial x_{4}^{2}}=0,\frac{\partial^{2}f_{4}}{\partial x_{1}\partial x_{4}}=0,\frac{\partial^{2}f_{5}}{\partial x_{1}^{2}}=0,\frac{\partial^{2}f_{5}}{\partial x_{1}\partial x_{2}}=0,\frac{\partial^{2}f_{5}}{\partial x_{2}^{2}}=0,\frac{\partial^{2}f_{5}}{\partial x_{3}\partial x_{2}}=0,\frac{\partial^{2}f_{5}}{\partial x_{4}\partial x_{2}}=0,\frac{\partial^{2}f_{5}}{\partial x_{3}^{2}}=0,\frac{\partial^{2}f_{5}}{\partial x_{1}\partial x_{3}}=0,\frac{\partial^{2}f_{5}}{\partial x_{4}\partial x_{3}}=0,\frac{\partial^{2}f_{5}}{\partial x_{4}^{2}}=0,\frac{\partial^{2}f_{5}}{\partial x_{1}\partial x_{4}}=0. \end{array}$$

Also,

(A.3) $$\begin{array}{c} \frac{\partial^{2}f_{2}}{\partial x_{2}\partial \beta^{*}}\left(E_{0},\beta^{*}\right)=1,\frac{\partial^{2}f_{1}}{\partial x_{2}\partial \beta^{*}}\left(E_{0},\beta^{*}\right)=-1,\frac{\partial^{2}f_{k}}{\partial x_{i}\partial \beta^{*}}\left(E_{0},\beta^{*}\right)=0,i\neq 2. \end{array}$$
